# Disease-modifying rdHSV-CA8* non-opioid analgesic gene therapy treats chronic osteoarthritis pain by activating Kv7 voltage-gated potassium channels

**DOI:** 10.3389/fnmol.2024.1416148

**Published:** 2024-07-17

**Authors:** Gerald Z. Zhuang, William F. Goins, Munal B. Kandel, Marco Marzulli, Mingdi Zhang, Joseph C. Glorioso, Yuan Kang, Alexandra E. Levitt, Konstantinos D. Sarantopoulos, Roy C. Levitt

**Affiliations:** ^1^Department of Anesthesiology, Perioperative Medicine and Pain Management, University of Miami Miller School of Medicine, Miami, FL, United States; ^2^Department of Microbiology and Molecular Genetics, University of Pittsburgh School of Medicine, Pittsburgh, PA, United States; ^3^Bascom Palmer Eye Institute, University of Miami Miller School of Medicine, Miami, FL, United States; ^4^John T. MacDonald Foundation Department of Human Genetics, University of Miami Miller School of Medicine, Miami, FL, United States; ^5^John P. Hussman Institute for Human Genomics, University of Miami Miller School of Medicine, Miami, FL, United States

**Keywords:** carbonic anhydrase-8, hyperalgesia, osteoarthritis, Kv7 voltage-gated potassium channels, KCNQ channels, neuropathic pain, persistent pain, gene therapy

## Abstract

Chronic pain is common in our population, and most of these patients are inadequately treated, making the development of safer analgesics a high priority. Knee osteoarthritis (**OA**) is a primary cause of chronic pain and disability worldwide, and lower extremity OA is a major contributor to loss of quality-adjusted life-years. In this study we tested the hypothesis that a novel JDNI8 replication-defective herpes simplex-1 viral vector (**rdHSV**) incorporating a modified carbonic anhydrase-8 transgene (CA8*) produces analgesia and treats monoiodoacetate-induced (**MIA**) chronic knee pain due to OA. We observed transduction of lumbar DRG sensory neurons with these viral constructs (vHCA8*) (~40% of advillin-positive cells and ~ 50% of TrkA-positive cells colocalized with V5-positive cells) using the intra-articular (**IA**) knee joint (**KJ**) route of administration. vHCA8* inhibited chronic mechanical OA knee pain induced by MIA was dose- and time-dependent. Mechanical thresholds returned to Baseline by D17 after IA KJ vHCA8* treatment, and exceeded Baseline (analgesia) through D65, whereas negative controls failed to reach Baseline responses. Weight-bearing and automated voluntary wheel running were improved by vHCA8*, but not negative controls. Kv7 voltage-gated potassium channel-specific inhibitor XE-991 reversed vHCA8*-induced analgesia. Using IHC, IA KJ of vHCA8* activated DRG Kv7 channels via dephosphorylation, but negative controls failed to impact Kv7 channels. XE-991 stimulated Kv7.2–7.5 and Kv7.3 phosphorylation using western blotting of differentiated SH-SY5Y cells, which was inhibited by vHCA8* but not by negative controls. The observed prolonged dose-dependent therapeutic effects of IA KJ administration of vHCA8* on MIA-induced chronic KJ pain due to OA is consistent with the specific activation of Kv7 channels in small DRG sensory neurons. Together, these data demonstrate for the first-time local IA KJ administration of vHCA8* produces opioid-independent analgesia in this MIA-induced OA chronic pain model, supporting further therapeutic development.

## Introduction

**Figure 1:**
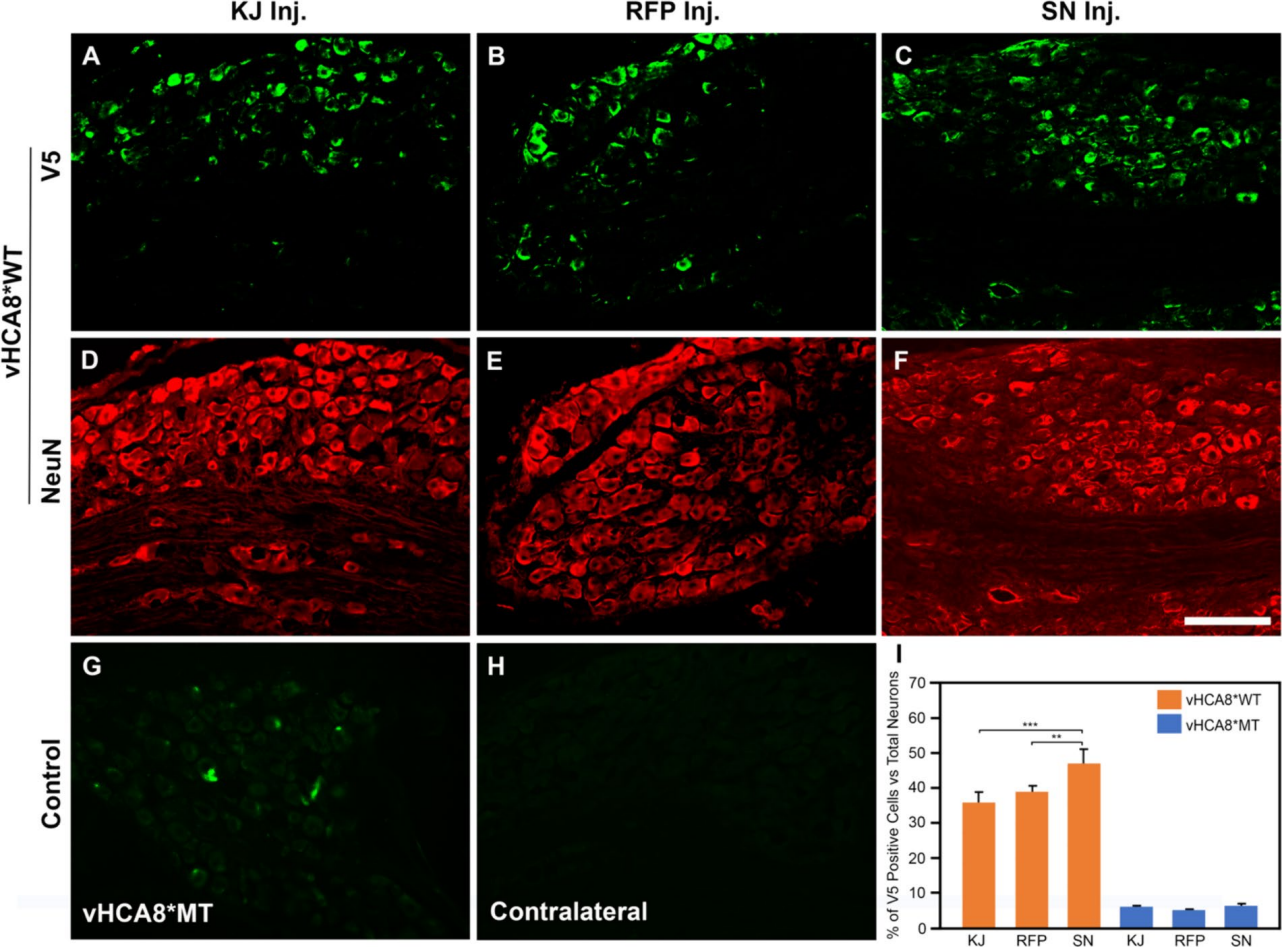
Neuronal DRG Transduction by vHCA8. IHC was performed with NeuN neuronal antigen expression, and V5 antibody to identify exogenous CA8* peptide expression on D14 after vHCA8*; using three different routes of administration including injection into the knee joint (KJ) **(A,D,I)**; rear foot pad (**RFP**) **(B,E,I)**; and sciatic nerve (SN) **(C,F–I)**. About 35–50% of ipsilateral lumbar 4–5 DRG neurons were V5-positive after vHCA8*WT treatment. The percentage of SN V5-positive neurons was higher than that in the KJ and RFP groups. Only about 5% of DRG neurons were V5-positive after vHCA8*MT treatment **(G)**. There was no V5-positive signal from contralateral DRG **(H)**. ImageJ version 1.54 was used to count stained cells. Groups were compared using IBM SPSS Statistics version 28 to do Student’s *t*-test, or ANOVA, followed by Fisher’s protected least significant difference (LSD) test, and these data are presented as mean ± SEM. Statistical significance was *p* < 0.05. Scale = 100 μm.

Chronic osteoarthritis (OA) pain, like most prevalent noncancer pain conditions, remains inadequately treated [Institute of Medicine (US) Committee on Advancing Pain Research, Care, and Education, 2011]. The impact of OA and other chronic pain disorders are enormous, costing the USA about $650 billion annually (McNicol et al., 2013; Busse et al., 2018). In the absence of suitable analgesic alternatives to treat chronic noncancer pain, an epidemic of opioid overuse, abuse, and life-threatening complications has occurred (Compton and Volkow, 2006; Paulozzi and Xi, 2008; Boudreau et al., 2009; Edlund et al., 2010a,b; Ostling et al., 2018). To address this unmet need, we set out to identify novel non-opioid analgesics to treat chronic OA pain. Prior transcriptome analysis demonstrate that dorsal root ganglia (**DRG**) carbonic anhydrase-8 (**Car8**, murine) expression regulates analgesic responses (Zhuang et al., 2015; Levitt et al., 2017). Car8 is an allosteric inhibitor of neuronal inositol trisphosphate receptor-1 (**ITPR1**). ITPR1 serves as a major endoplasmic reticulum (**ER**) calcium release channel, converting and amplifying inositol trisphosphate (**IP3**) responses into intracellular calcium signaling (Berridge, 1993). Car8 selectively binds to the modulatory domain of ITPR1 and specifically inhibits its activation by phosphorylation (**pITPR1**) (Hirota et al., 2003), which decreases ER calcium release and reduces cytoplasmic free calcium, essential to the regulation of neuronal excitability. *Waddle (wdl −/−)* mice demonstrate decreased mechanical withdrawal thresholds (*p* < 0.01; Figure 1A) and thermal withdrawal latencies, increased pITPR1, and cytoplasmic free calcium at baseline due to a *Car8* deletion (Zhuang et al., 2015). DRG Car8 complementation was necessary and sufficient to reverse these abnormalities in wdl −/− mice. We later showed that modified human carbonic anhydrase-8 (CA8*) retained the same functions, despite the significant variation in peptide sequence between species (Zhuang et al., 2015, 2018; Upadhyay et al., 2019, 2020). To address the hypothesis that CA8* represents a novel non-opioid analgesic that could be delivered locally, we administered CA8* via sciatic nerve injection using adeno-associated virus-based **(AAV)** gene therapy to mice (Zhuang et al., 2018). This AAV-CA8* gene therapy vector transduced mouse DRG after sciatic nerve injections to produce profound long-lasting analgesia (equivalent >100 mg of oral morphine in 60 kg adult for more than 4 weeks) to treat chronic pain in various models (Fu et al., 2017; Zhuang et al., 2015, 2018; Upadhyay et al., 2019, 2020). Unlike current local anesthetics, CA8* related analgesia occurred without motor blockade, complete sensory loss, or clinical pathology (Zhuang et al., 2015, 2018; Fu et al., 2017; Levitt et al., 2017; Upadhyay et al., 2019). Despite the demonstrated fundamental role of CA8 in regulating intracellular calcium signaling, our understanding of how CA8 regulates neuronal excitability to produce analgesia remains unknown.

One potential mechanism is via opening of Kv7 voltage-gated potassium channels. Kv7 channels are the only known neuronal potassium channels that are activated by lower cytoplasmic calcium to produce M-currents (I_M_) through calmodulin-dependent and -independent mechanisms (Selyanko and Brown, 1996a; Gamper and Shapiro, 2003; Delmas and Brown, 2005; Burgoyne, 2007; Kosenko and Hoshi, 2013). I_M_ regulate neuronal excitability and produce analgesia by prolonging neuronal AHP, which restricts the firing of action potentials (Lombardo and Harrington, 2016; Wu et al., 2017) and the propagation of afferent nociceptive signals. Kv7-mediated I_M_ are abundant in small DRG neurons comprised of Kv7.2, Kv7.3 and Kv7.5 subunits (Passmore et al., 2003). Apparently, DRG expression of these channel subunits is unchanged by spinal cord injury (Wu et al., 2017). Previously, Kv7.2 and Kv7.3 phosphorylation sites were identified as critical to channel regulation by protein kinase A, protein kinase C, and src tyrosine kinase (Surti et al., 2005). Using mass spectrometry, mutagenesis and electrophysiological studies, Surti et al. (2005) reported a mechanism of critical channel inhibition by specific phosphorylation of Kv7.2 at Threonine 217 and Kv7.3 at Threonine 246, which are the most abundant Kv7 channel subunits in peripheral sensory neurons. These sites are located within the S4 –S5 intracellular loop. Kv7 voltage-gated potassium channel openers (e.g., flupirtine, retigabine) are well-known to produce non-opioid-based analgesia in a variety of animal models and human chronic pain conditions (McMahon et al., 1987; Mastronardi et al., 1988; Blackburn-Munro and Jensen, 2003; Erichsen et al., 2003; Passmore et al., 2003; Munro and Dalby-Brown, 2007; Wulff et al., 2009; Perdigoto et al., 2018). However, despite their utility in treating various forms of chronic noncancer pain, all Kv7 channel openers were removed from the market due to adverse events related to their oral use and systemic exposure (Konishi et al., 2018; Perdigoto et al., 2018). Nonetheless, Kv7 channels remain important analgesic targets. Recently, we showed vHCA8* prolongs the afterhyperpolarization (AHP) by selective activation of Kv7 channels to produce I_M_ currents (Kandel et al., 2024), shown to exert powerful control of neuronal excitability (Brown and Passmore, 2009). These findings mechanistically link vHCA8*, ITPR1 inhibition, decreased intracellular calcium ([Ca2+]i), with Kv7 channel activation. Therefore, we speculate Kv7 channel activation by CA8*-mediated reduction of cytoplasmic free calcium contributes to analgesia observed in animal models.

A major limitation of AAV strains used in our prior studies was their limited potential to transduce DRG neurons except after direct intra-neural injections (Chan et al., 2017; Zhuang et al., 2018; Chakrabarti et al., 2020). In contrast, HSV-based gene therapy has the potential to transduce DRG neurons after IA KJ injections (Oligino et al., 1996; Lu et al., 2008; Wu et al., 2013), commonly used for short-term localized treatment of chronic OA KJ pain. Direct IA KJ route of administration is appealing because it is expected to greatly limit peripheral nervous system (**PNS**) exposure and potential toxicity related to gene therapy, as compared to intra-neural, direct DRG or systemic injections. Nonetheless, an important consideration regarding direct joint injections is the preservation of joint cartilage (LaBranche et al., 2017). The HSV gene therapy used in our current study is based on JDNI8, which are highly defective non-replicating viral vectors that are deleted for functional expression of all the viral immediate early (**IE**) genes (Miyagawa et al., 2015, 2017; Verlengia et al., 2017). These replication defective (rd)HSV vectors provide an efficient delivery system to the PNS that selectively establishes natural lifelong latency within infected neurons following retrograde transport of viral particles to the nerve cell body in DRG. Together these viral modifications provide a non-cytotoxic disease-free vector capable of long-term episomal maintenance in sensory neuron somata with robust long-lived transgene expression (Verlengia et al., 2017). These viral vectors provide no viral antigen targets for immune effector cells and, consequently, are much less likely to produce exaggerated immune responses observed with other gene therapy vectors (Miyagawa et al., 2015, 2017; Verlengia et al., 2017).

The primary goal of the present study was to make use of this novel gene therapy system to test the hypothesis that vHCA8* vector delivery and expression of a human CA8* transgene as a CA8 peptide variant can treat chronic pain by activating Kv7 channels to produce long-lasting analgesia and anti-hyperalgesia in a well-known mouse OA model (Chandran et al., 2009; Vonsy et al., 2009; Stevenson et al., 2011; Nagase et al., 2012; Okun et al., 2012). This OA model consists of direct IA KJ administration of MIA to induce prolonged painful mechanical allodynia (Nagase et al., 2012). Our results show disease-modifying analgesia following CA8* transgene expression in the relevant ganglia by several relevant routes of administration, including the clinically preferred IA KJ injection. The mechanism of action is consistent with CA8* activation of the Kv7 voltage-gated potassium channels and the specificity of the treatment was confirmed using negative controls and drug-mediated (XE-991) selective antagonism of Kv7 channels *in vitro and in vivo*.

## Results

### vHCA8* transduces lumbar DRG neurons via different routes of administration

For these studies, we created JDNI8-CAGp-CA8WT-V5-T2A-GFP (vHCA8*WT) and JDNI8-CAGp-CA8MT-V5-T2A-GFP (vHCA8*MT) constructs containing the wildtype CA8-201 (WT) and CA8 mutant (MT) [CA8-201 with S100P null point mutation (Türkmen et al., 2009)] cDNAs modified with a V5 tag. The CA8*MT represents a rigorous negative control because this vector and transgene are identical in every way to vHCA8*WT except for this point mutation that produces a nearly complete loss of CA8* cellular protein associated with rapid proteasome-mediated degradation (Türkmen et al., 2009). The vHCA8*WT vector is depicted in Supplementary Figure S1A. These transgenes were inserted downstream of the **CAG** promoter (**C** cytomegalovirus early enhancer element; **A** the promoter region, first exon and the first intron of the chicken beta-Actin gene; and **G** the splice acceptor of the rabbit beta-Globin gene) that is known to be active in a broad array of cell types including neurons (Zhuang et al., 2018).

To verify that these vectors produce the proper sized CA8* protein product in rat primary DRG neurons in culture, vector-infected DRG cell lysates harvested 2 days post-infection were employed in western blot analyses using the CA8, V5 tag, and β-actin antibodies (Supplementary Figure S1B). We demonstrated that the vHCA8*WT vector yielded high levels of the correct sized CA8* product detected with both V5 and CA8 antibodies, while the vHCA8*MT negative control vector expressed greatly reduced levels of the CA8* protein in comparison to the β-actin loading control, similar to that seen previously with the AAV-CA8*WT and -MT vectors (Zhuang et al., 2018).

**Figure 2:**
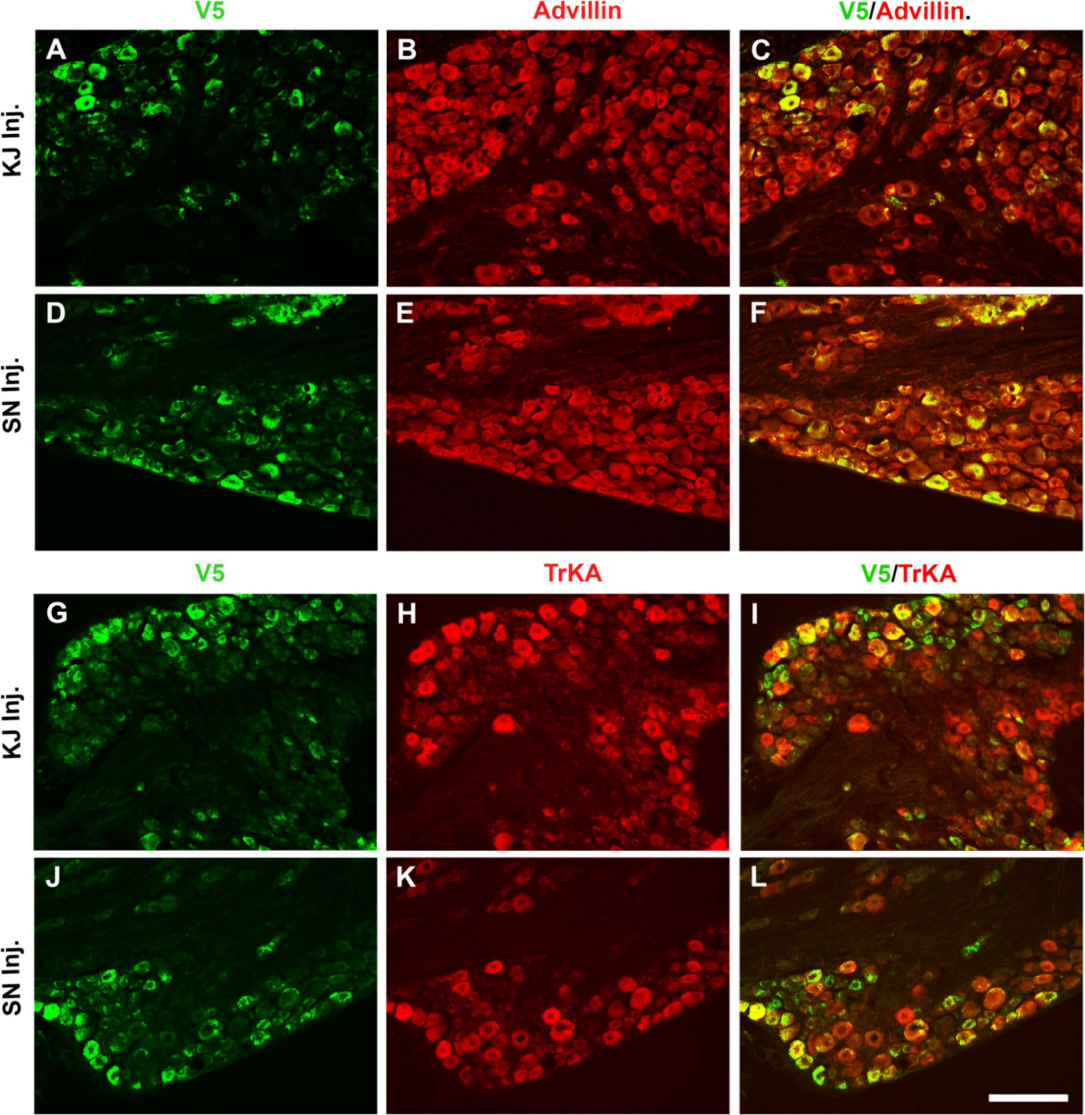
Infection after sciatic nerve or intra-articular injection of vHCA8*WT shows selective CA8*WT expression in small sensory DRG neurons. KJ were injected with vHCA8*WT on D0. D14 after KJ or SN administration of vHCA8*WT high-dose (1E6 PFU), L4-5 DRG were harvested. DRG shown were double immunofluorescence stained with anti-V5, pan-sensory neuronal maker anti-advillin or anti-TrkA (smaller primary afferents). The percentage of V5-positive cells colocalized with advillin-positive neurons is 35.3 ± 4.2 in the IA KJ injected group **(A–C)** and 43.9 ± 4.8 in the SN injected group **(D–F)**, respectively. The percentage of TrkA-positive cells colocalized with V5 is 45.6 ± 3.9 in the IA KJ injected group **(G–I)** and 54.2 ± 6.2 in the SN injected group, respectively **(J–L)**. ImageJ version 1.54 was used to count stained cells. *N* = 4 animals per group. Scale = 100 μm.

To directly compare the ability of our HSV constructs to transduce murine DRG neurons *in vivo* using different clinically relevant routes of administration, we examined ipsilateral and contralateral L4 and L5 DRG expression of exogenous CA8* using V5-immunohistochemistry (**IHC**). D14 after injections with vHCA8*WT or vHCA8*MT using the IA KJ (Figures 1A,D), sciatic nerve (**SN**) (Figures 1C,F), or rear footpad (**RFP**) (Figures 1B,E) routes of injection. CA8*WT expression was higher in DRG after SN injections (47.3 ± 4.1%), compared to KJ (35 ± 3.2%) or RFP (39.1 ± 3.7%) (Figure 1I). In contrast, ~5% of neurons were V5-positive in the ipsilateral DRG after injections with vHCA8*MT (Figures 1G,I). There were no V5 positive neurons observed from contralateral lumbar DRG (Figure 1H), again demonstrating the lack of replication seen when using these rdHSV vectors. Remarkably, despite the CAG promoter used, which expresses in all cells, CA8*WT (exogenous CA8*) expression co-localized largely with somatosensory DRG neurons as ascertained with double IHC staining. After KJ injections DRG CA8*WT expression showed co-localization of V5- with advillin-positive neurons (a marker for pan-sensory neurons) (Figures 2A–C), or V5- and TrkA-positive DRG neurons (generally small sensory neurons expressing the high-affinity NGF receptor) (Figures 2G–I). DRG CA8*WT neuronal expression after SN injections also showed co-localization of V5- and advillin-positive neurons (Figures 2D–F), or V5- and TrkA-positive DRG neurons (Figures 2J–L). The percentage of advillin-positive cells colocalizing with V5-positive cells was 35.3 ± 4.2 in the IA KJ group and 43.9 ± 4.8 in the SN group, respectively. The percentage of TrkA-positive cells colocalizing with V5-positive cells was 45.6 ± 3.9 in the IA KJ group and 54.2 ± 6.2 in the SN group, respectively, 2 weeks after transduction of lumbar DRG sensory neurons with vHCA8* viral particles.

### Dose-dependent vHCA8* reversal of MIA-induced weight-bearing reduction

**Figure 3:**
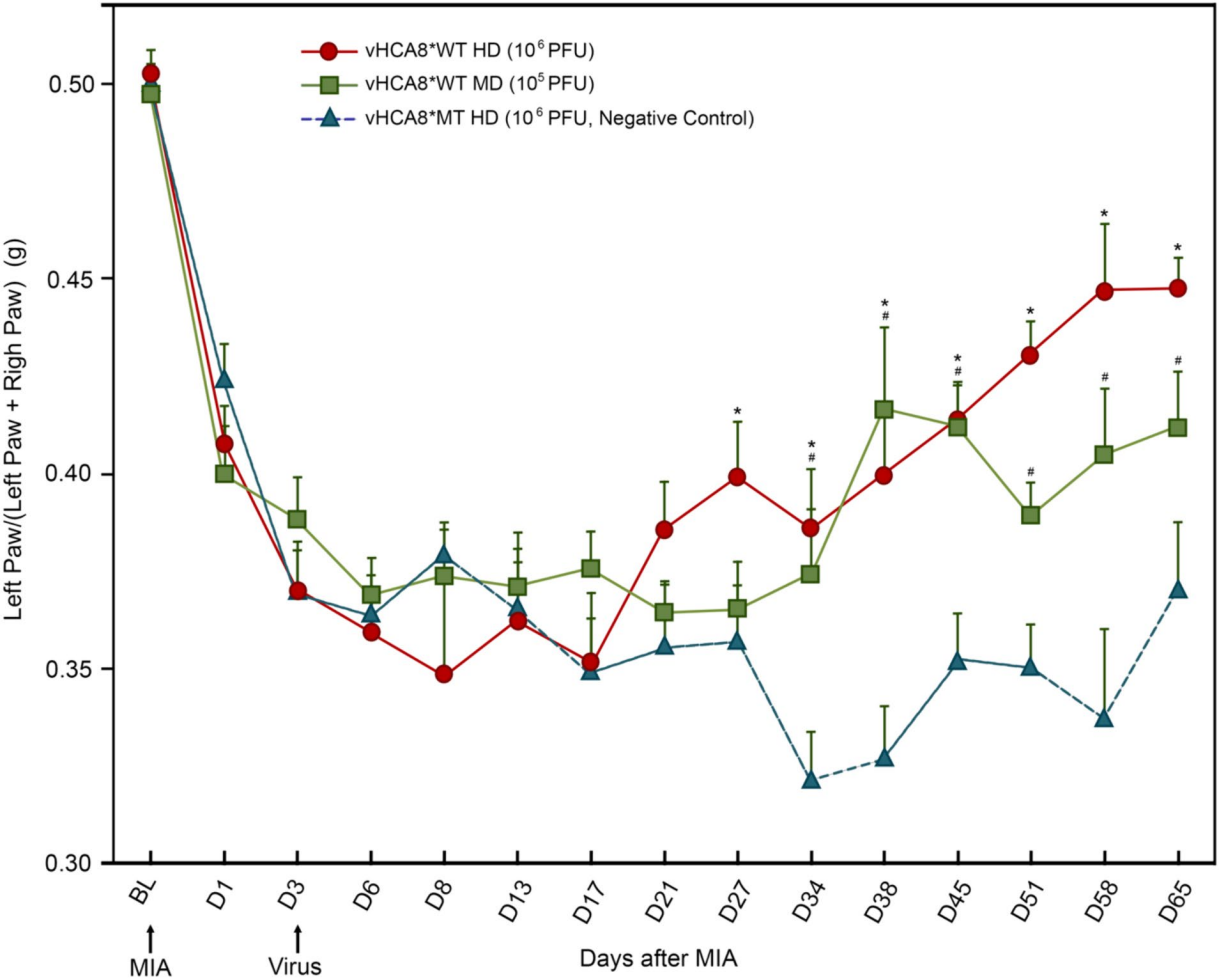
vHCA8*WT reverses MIA-OA-induced decreases in weight-bearing. Male C57BL/6 mice underwent IA KJ injection with 1 mg of monosodium iodoacetate (MIA) on D1 (after Baseline was established) producing hyperalgesia in all mice. Weight-bearing was assessed at about the same time each day as weight (grams) of ipsilateral (treated) limb/weight of ipsilateral limb + weight of contralateral limb (untreated control). On D3 mice were treated with IA KJ injections of either vHCA8*WT at the High-Dose (HD) (1E6 PFU), Mid-Dose (MD) (1E5 PFU) or vHCA8*MT High-Dose (HD) (1E6 PFU) (virus arrow) after weight-bearing assessment. Weight-bearing as measured (grams) dropped starting on D1 after MIA injection in all mice. Weight-bearing increased significantly in mice treated with vHCA8*WT-HD or vHCA8*WT-MD on D27 and D34, respectively, as compared to mice treated with vHCA8*MT-HD. * *p* < 0.05 for vHCA8*WT-HD vs. vHCA8*MT-HD, # *p* < 0.05 for vHCA8*WT-MD vs. vHCA8*MT-HD. Data were analyzed for statistical significance between different time points for each group, as compared to Baseline, by one-way analysis of variance (ANOVA) followed by Fisher’s LSD post-hoc test. Repeated measure two-way ANOVA analyses found significant differences in weight distribution between left (OA) and right (contralateral control) hind limbs across all time points [*F*(14, 196) = 9.637, *p* = 3.898E-16]. *N* = 10 per group.

**Figure 4:**
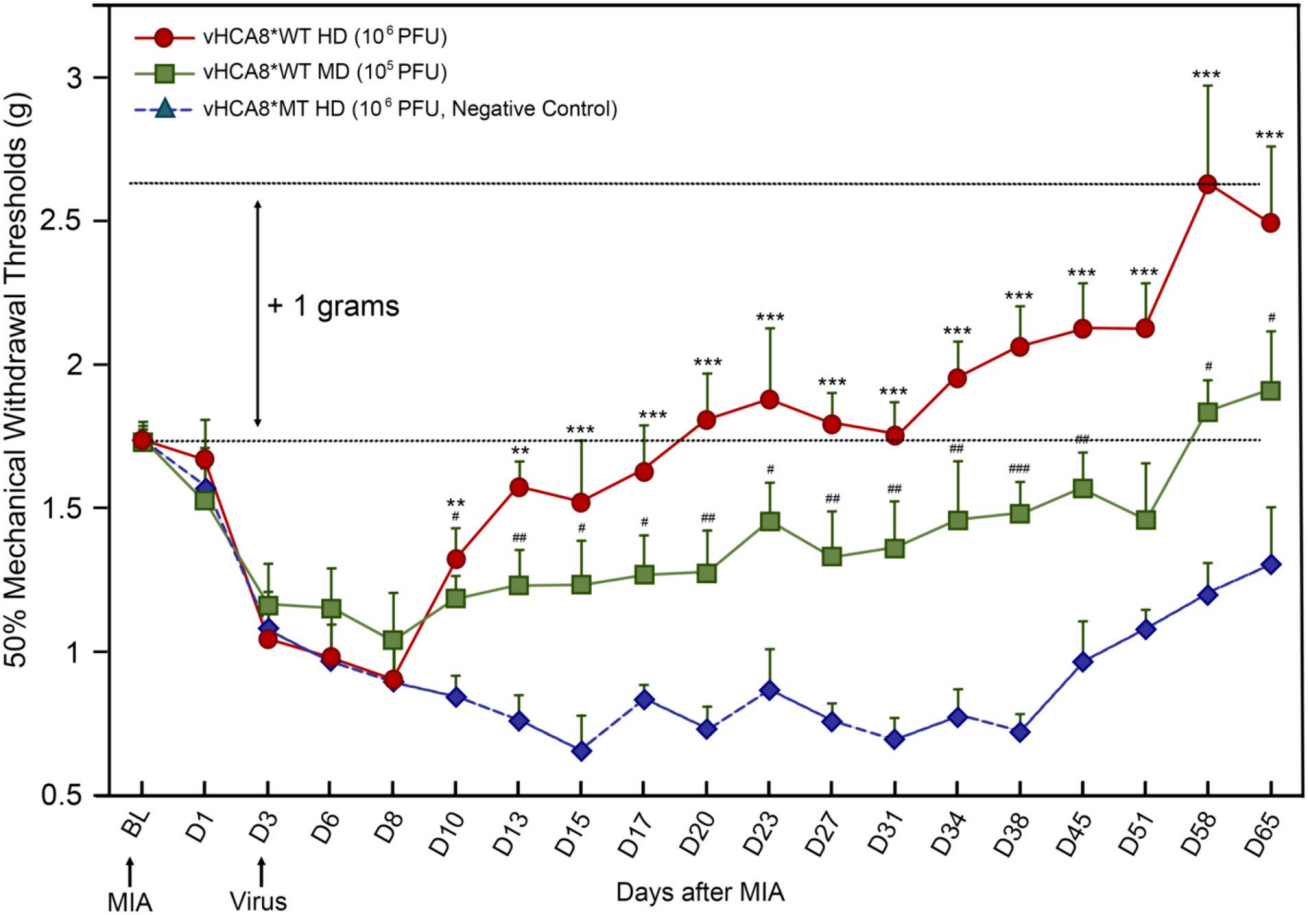
vHCA8*WT produces analgesia and inhibits MIA-OA-induced mechanical hyperalgesia. Male C57BL/6 mice were injected with vHCA8*WT at the High-Dose (HD) (1E6 PFU), Mid-Dose (MD) (1E5 PFU) and compared with vHCA8*MT High-Dose (HD) (1E6 PFU) using the KJ route of administration (IA KJ). IA KJ injection of monosodium iodoacetate (MIA) on D1 after Baseline (arrow) produced hyperalgesia in all mice. KJ injections were performed with vHCA8*WT or MT virus after testing mechanical withdrawal responses on D3 after MIA (arrow). Mechanical thresholds in vHCA8*WT dose groups differed from the vHCA8*MT negative controls as shown through D65 after MIA. vHCA8*MT negative control failed to return to Baseline through D65. vHCA8*WT-HD and vHCA8*WT-MD demonstrated anti-hyperalgesia starting on D10. vHCA8*WT-HD exceeded Baseline starting on D23 (analgesia) and vHCA8*WT-MD exceeded Baseline starting on D58 (*N* = 10 per group) (One-way ANOVA). * *p* < 0.05 ** *p* < 0.01, and *** *p* < 0.001 for vHCA8*WT-HD vs. vHCA8*MT-HD, # *p* < 0.05, ## *p* < 0.01, and ### *p* < 0.001 for vHCA8*WT-MD vs. vHCA8*MT-HD. Repeated measures two-way ANOVA: Significant differences in mechanical threshold were found between groups [*F*(2,27) = 146.5, *p* = 3.19E-15] and across all time points [*F*(18,486) = 10.94, *p* = 2.25E-26]. There were also significant differences in interactions between time points and groups [*F*(36,486) = 3.16, *p* = 8.61E-09].

IA left KJ injection of 1 mg of MIA induced a significant reduction in weight-bearing in all groups starting from D1 as assessed as weight in grams of ipsilateral (treated) limb/weight of ipsilateral (treated) + weight of contralateral limb (untreated control). Three days after IA KJ MIA injection, the ipsilateral KJ was again injected with either vHCA8*WT at one of two doses, high-dose (HD, 1E6 PFU); or mid-dose (MD, 1E5 PFU) or high-dose vHCA8*MT (HD, 1E6 PFU) (Figure 3). After the initial drop, weight-bearing significantly improved as compared to vHCA8*MT-HD treated mice, when treated with vHCA8*WT-HD starting D27 and D34 in mice treated with vHCA8*WT-MD and persisted to D65 in both groups. These weight-bearing improvements corresponded with anti-hyperalgesia and analgesia (increase above baseline in mechanical withdrawal thresholds in the vHCA8*WT-HD groups (Figure 4). Data were analyzed for statistical significance between different time points for each group, as compared to Baseline, by one-way analysis of variance (ANOVA) followed by Fisher’s LSD post-hoc test. Repeated measures two-way ANOVA analyses found significant differences in weight distribution between left (OA) and right (contralateral controls) hind limbs across all time points [*F*(14, 196) = 9.637, *p* = 3.898E-16]. But there were no significant differences between groups [*F*(1, 14) = 139.24, *p* = 0.745]. There were also no significant interactions between time points and groups [*F*(14, 196) = 0.803, *p* = 0.666].

Additional studies were run to test the impact of IA KJ MIA treatment on voluntary activity as assessed with AVWR (Automated Voluntary Wheel Running) in male C57BL/6 mice. Baseline voluntary wheel running behaviors were collected for one hour of free access to the activity wheel after 4 habituation sessions. IA KJ injection of 1 mg of MIA induced a significant reduction in voluntary wheel running (Supplementary Figure S2). Three days after IA KJ MIA injection, the ipsilateral KJ was again injected with either vHCA8*WT (HD 1E6 PFU) or vHCA8*MT (HD 1E6 PFU). After the initial drop in both groups, voluntary wheel running distance improved in vHCA8*WT-HD as compared to vHCA8*MT on D9-D11, with both groups improving to Baseline by D13.

### Dose-dependent vHCA8* analgesia and reversal of MIA-induced hyperalgesia after knee joint injection

Three days after left KJ MIA administration, the KJ was again injected with either vHCA8*WT or vHCA8*MT (negative control). Mechanical thresholds in the IA KJ injected vHCA8*WT dose groups were significantly higher than those in the vHCA8*MT-HD group starting on D10 (Figure 4). Mechanical thresholds for the IA KJ injected vHCA8*MT-HD group never returned to the Baseline through D65. In contrast, the vHCA8*WT-HD treated group was significantly higher than vHCA8*MT HD negative controls and exceeded Baseline (analgesia) after D20, which persisted to D65. Mechanical thresholds in the vHCA8*WT-MD (1E5 PFU) treated group were significantly higher than vHCA8*MT-HD after D10 but only exceeded Baseline (analgesia) after D58. Data were analyzed for statistical significance between different time points for each group, as compared to Baseline, by one-way analysis of variance (ANOVA) followed by Fisher’s least significance difference (LSD) post-hoc test. Repeated measures two-way ANOVA found significant differences in mechanical thresholds between groups [*F*(2,27) = 146.5, *p* = 3.19E-15] and across all time points [*F*(18,486) = 10.94, *p* = 2.25E-26]. There were also significant differences in interactions between time points and groups [*F*(36,486) = 3.16, *p* = 8.61E-09].

### Kv7 specific inhibitor XE-991 reverses vHCA8* analgesia

**Figure 5:**
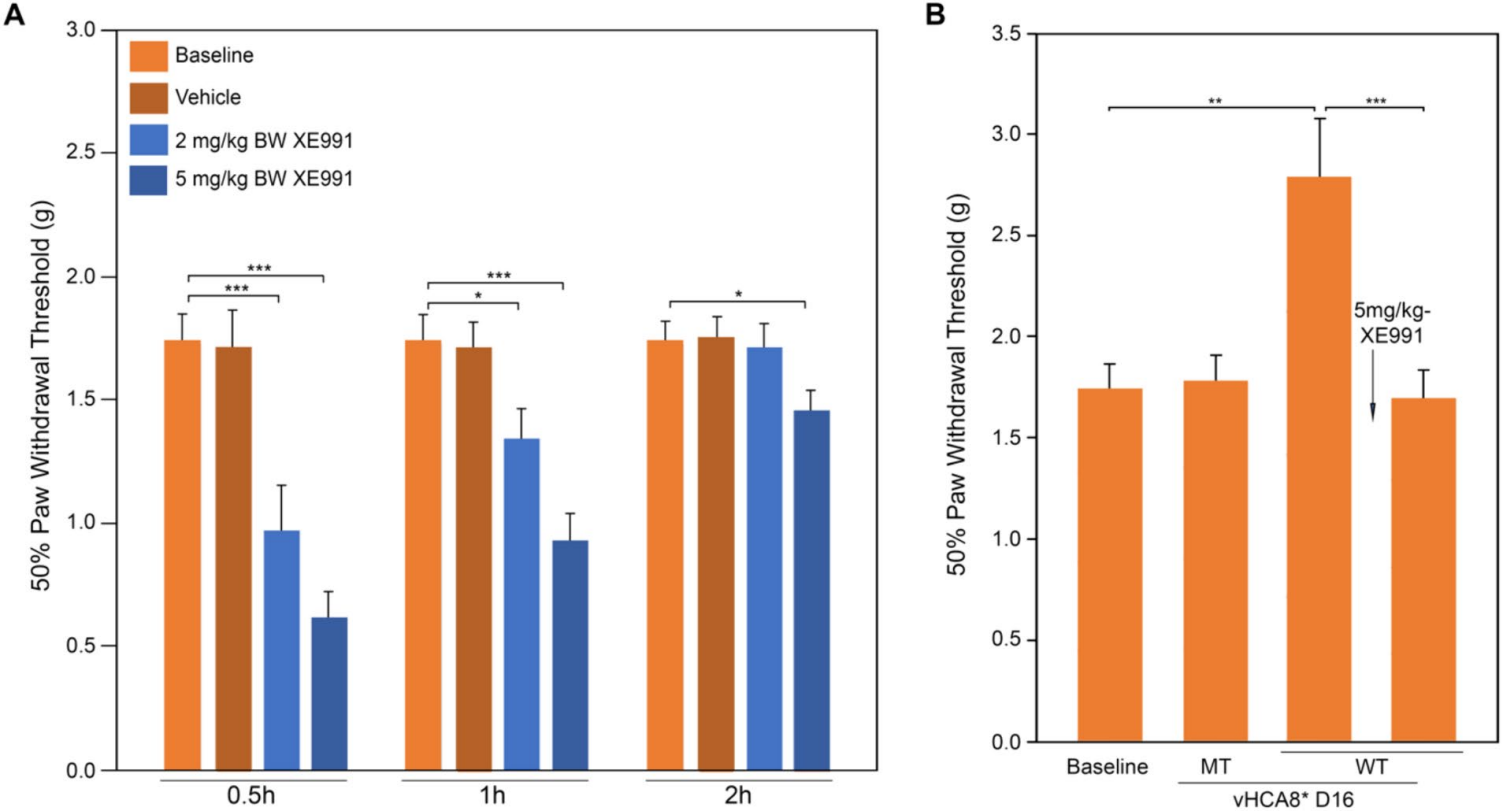
Kv7 specific antagonist inhibits CA8*WT analgesia in response to MIA-induced chronic OA. Kv7-specific antagonist XE-991 induced mechanical hyperalgesia at Baseline in a dose-dependent and time-dependent manner (no MIA). XE-991 (2 mg/kg and 5 mg/kg produced hyperalgesia lasting up to 2 h **(A)**. vHCA8*WT administration (SN injection) induced analgesia at D16 and XE-991 (5 mg/kg i.p.) reversed the analgesic effects of vHCA8*WT acutely to baseline **(B)** (*N* = 8 per group). * *p* < 0.05; ** *p* < 0.01; *** *p* < 0.001; one-way ANOVA).

The effects of the Kv7-specific antagonist XE-991 on vHCA8*WT analgesia in naïve mice are shown in Figure 5. XE-991 reduced mechanical thresholds in a time- and dose-dependent fashion in naïve mice compared to Baseline (pre-treatment) or vehicle control (Figure 5A). Mechanical thresholds returned to Baseline in approximately 2 h after intraperitoneal (IP) administration of XE-991 low-dose groups (e.g., 2 and 5 mg/kg) (data Figure 5A). In separate experiments, mechanical thresholds increased above baseline (analgesia) on D16 after SN injection of vHCA8*WT, but vHCA8*MT had no effect (Figure 5B). Mechanical thresholds returned to Baseline at 30 min after IP after IP administration of XE-991 (5 mg/kg). These data are consistent with vHCA8*WT-induced analgesia and anti-hyperalgesia being mediated selectively by Kv7 channel activation.

### Reduction of DRG pKv7 by IA KJ injections of vHCA8*WT

**Figure 6:**
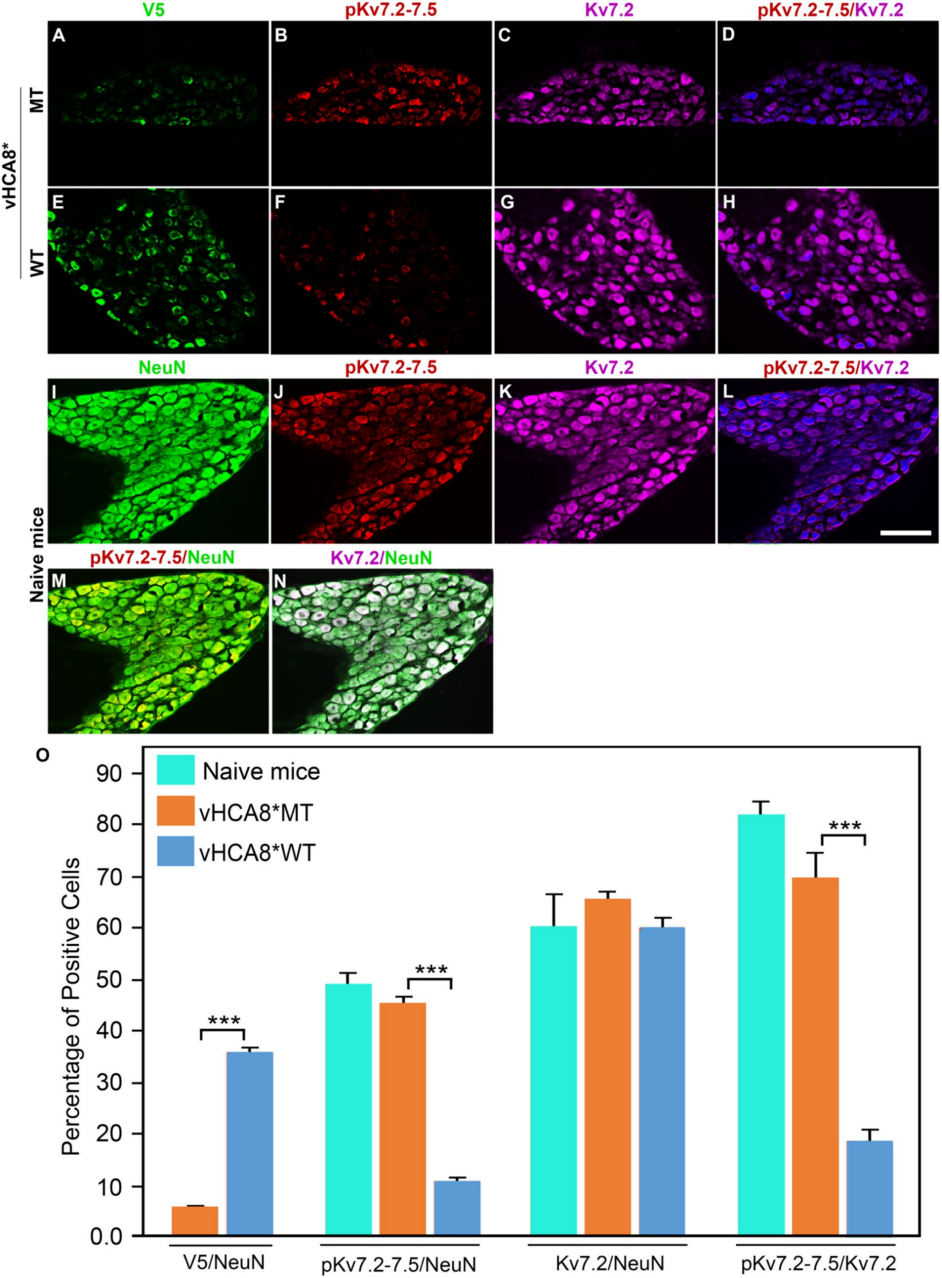
Impact of KJ vHCA8* injections on Kv7.2–7.5 activation assessed in DRG using IHC. The left KJ of naïve mice was injected with vHCA8*MT or vHCA8*WT. **(A)** Low levels of V5-staining (5.7%) are seen after KJ IA injection of vHCA8*MT-HD (10E6 PFU). **(B–D,O)** There is no effect on DRG pKv7.2–7.5/NeuN, pKv7.2–7.5/Kv7.2 expression ratios in vHCA8*MT-HD (1E6 PFU) treated animals; **(E)** vHCA8*WT-HD (1E6 PFU) treated animals show higher levels of V5-staining (36.0%). **(F–H,O)** vHCA8*WT-HD treated mice show reduced pKv7.2–7.5/NeuN, pKv7.2–7.5/Kv7.2 expression ratios compared to vHCA8*MT-HD treated animals. The ratio of Kv7.2 to NeuN was unchanged by vHCA8*WT or vHCA8*MT KJ injections compared to naïve mice. Results from naïve mice **(I–N,O)** are shown after staining for **(I)** NeuN, **(J)** pK7.2–7.5, **(K)** Kv7.2, **(L)** pKv7.2–7.5/Kv7.2, **(M)** pKv7.2–7.5/NeuN, **(N)** Kv7.2/NeuN. *N* = 4 mice per group. Scale = 100 μm, *** *p* < 0.001; one-way ANOVA.

**Figure 7:**
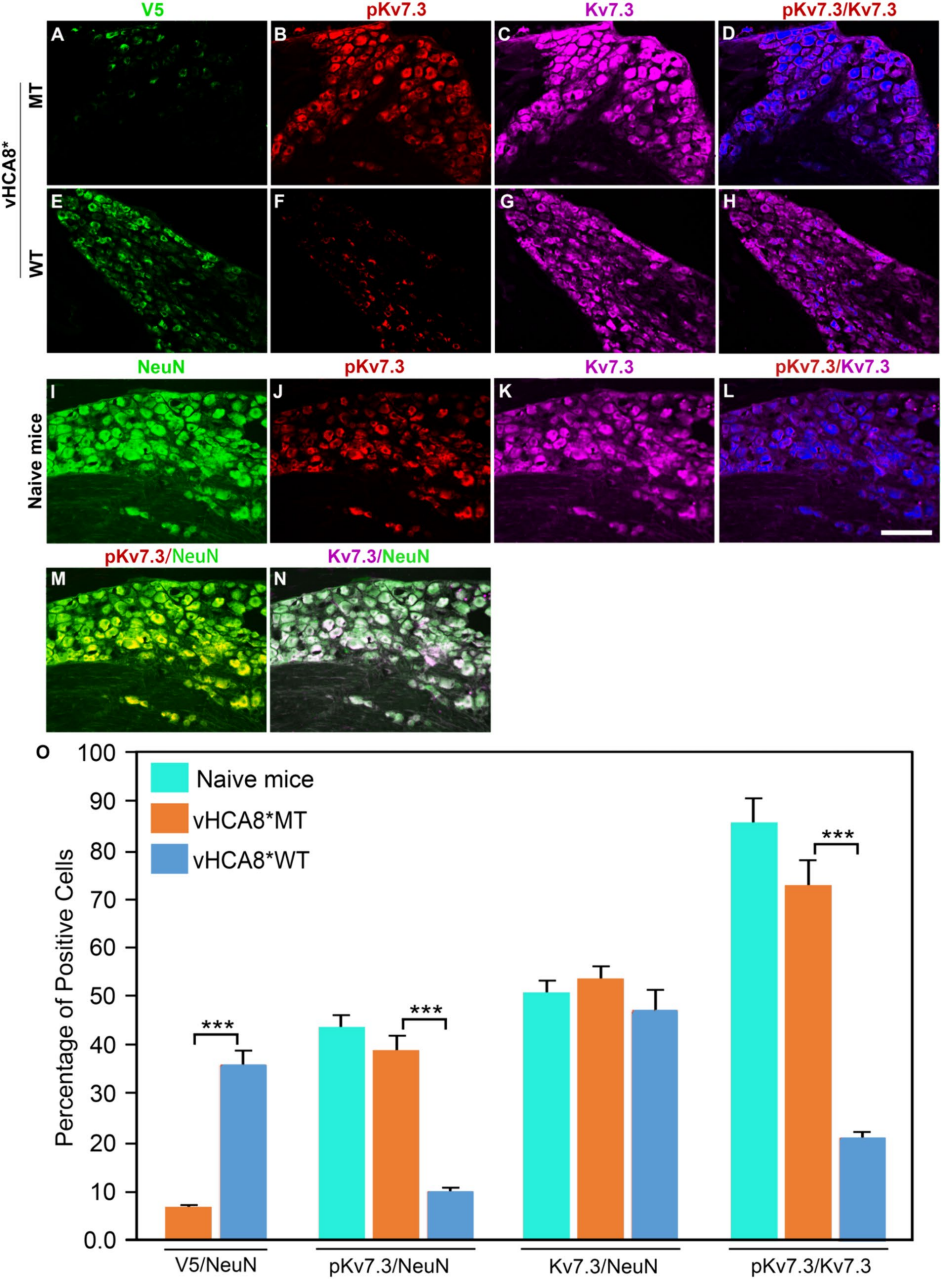
Impact of KJ vHCA8* injections on Kv7.3 activation assessed in DRG using IHC. The left KJ of naïve mice was injected with vHCA8*MT or vHCA8*WT. **(A)** V5-staining was lower after KJ injection of vHCA8*MT-HD (1E6 PFU) (6.8%) as compared to **(E)** vHCA8*WT-HD (1E6 PFU) (35.9%). **(B–D,O)** There is no effect on DRG pKv7.3/NeuN, pKv7.3/Kv7.3 expression ratios in vHCA8*MT-HD (1E6 PFU) treated animals; **(F–H,O)** KJ injection with vHCA8*WT-HD reduced pKv7.3/NeuN, pKv7.3/Kv7.3 expression ratios compared to vHCA8*MT-HD treated animals. The ratio of Kv7.3 to NeuN was unchanged by vHCA8*WT-HD or vHCA8*MT-HD KJ injections compared to naïve mice. Results from naïve mice **(I–N,O)** are shown after staining for **(I)** NeuN, **(J)** pK7.3, **(K)** Kv7.3, **(L)** pKv7.3/Kv7.3, **(M)** pKv7.3/NeuN, and **(N)** Kv7.3/NeuN. *N* = 4 mice per group. Scale = 100 μm, *** *p* < 0.001; one-way ANOVA.

To explore whether vHCA8* affects *in vivo* Kv7 channel activity, which is negatively regulated by channel phosphorylation (Surti et al., 2005), vHCA8* was injected into the left KJ and on D14 DRG samples were collected and analyzed using immunofluorescence. Representative IHC sections stained for V5, pKv7.2–7.5, pKv7.3, Kv7.2, and Kv7.3 are shown in Figures 6, 7. The percentage of pKv7-positive cells were normalized to NeuN-, Kv7.2-, or Kv7.3-positive neurons (ratios) (Figures 6, 7). DRG V5 expression (exogenous CA8*) was barely detectable after vHCA8*MT injections (Figures 6A, 7A). For the vHCA8*MT IA KJ treated group, DRG pKv7.2–7.5/Kv7.2 (Figures 6A–F,O) and DRG pKv7.3/Kv7.3 (Figures 7A–D,O) did not differ from naïve animals (Figures 6I–L,O, 7I–L,O, respectively). After vHCA8*WT KJ injection, the ratio of DRG pKv7.2–7.5/NeuN and pKv7.2-5/Kv7.2 and pKv7.3/NeuN and pKv7.3/Kv7.3 were significantly reduced, as compared to after treatment with vHCA8*MT negative control or naïve mice (Figures 6E–H,M,O, 7E-H,M,O, respectively). DRG Kv7.2/NeuN and Kv7.3/NeuN were unaffected by vHCA8*WT or vHCA8*MT (Figures 6N,O, 7N,O, respectively), demonstrating rdHSV infection had no significant impact on Kv7 channel expression. About 35% of DRG neurons were V5-positive after vHCA8*WT treatment (Figures 6E,O, 7E,O) (V5 tag was fused to CA8 C-terminal region).

### vHCA8*WT reverses XE-991 stimulated pKv7 in differentiated SH-SY5Y cells

SH-SY5Y cells after RA differentiation expressed high levels of CA8*WT peptide (using V5-tag) on western blots after infection with vHCA8*WT (Supplementary Figure S3A), as compared to CA8*MT (V5-tag) after vHCA8*MT infection, or vehicle control (Supplementary Figure S3A) at Baseline. The expression of Kv7 channels in SH-SY5Y cells differed in response to retinoic acid (RA) differentiation (Supplementary Figures S3B,B’). Kv7.2 expression increased with RA differentiation through D9, while Kv7.3 expression peaked about D4 (Supplementary Figure S3B’). Kv7.5 expression decreased with RA differentiation and was not studied in SH-SY5Y cells, any further. Because both Kv7.2 and Kv7.3 subunits are prominently expressed in DRG small sensory fibers (Yu et al., 2018), we focused our further analyses on Kv7.2 and Kv7.3 channels in SH-SY5Y on D9 after RA-differentiated.

**Figure 8:**
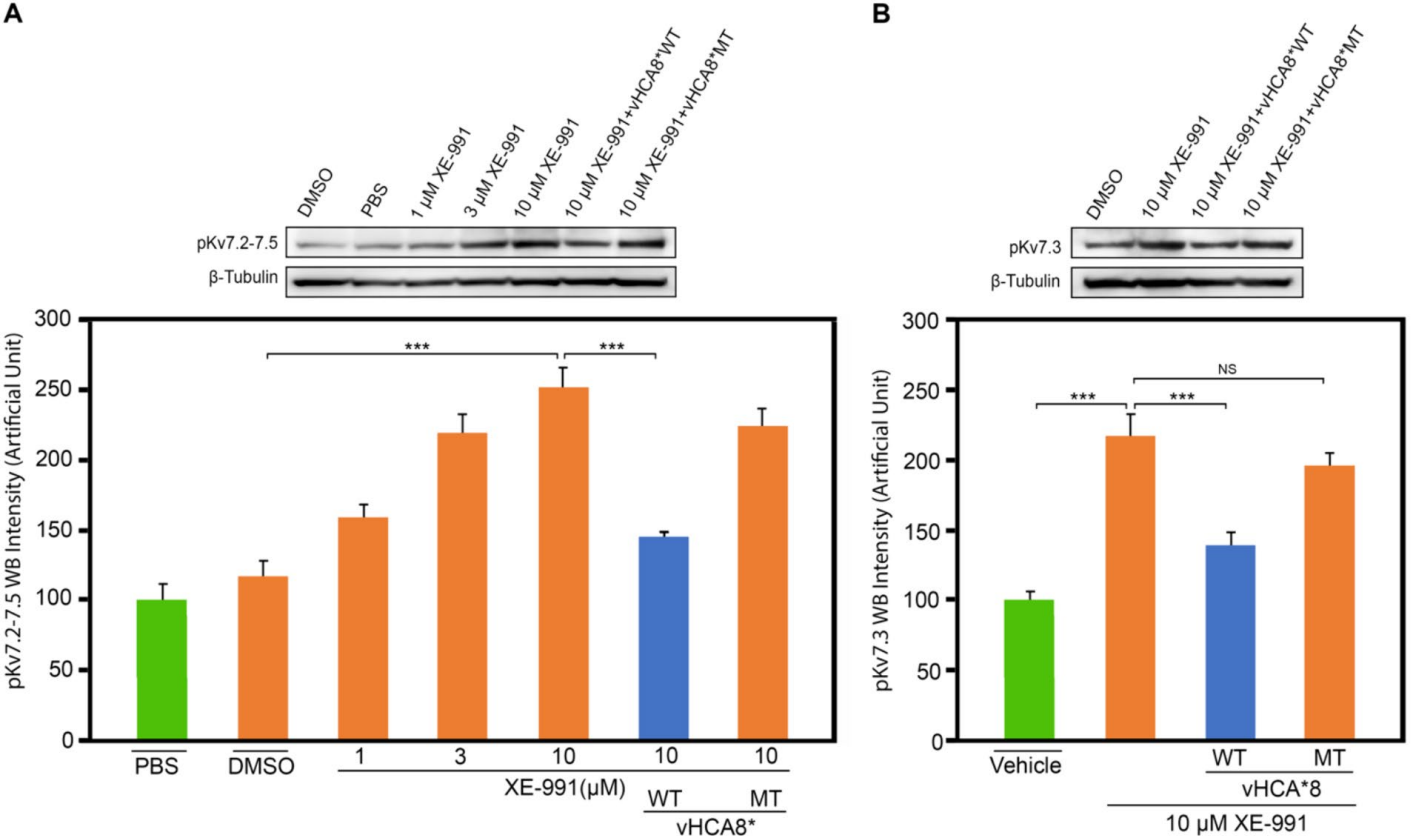
vHCA8*WT inhibits XE-991-induced Kv7 phosphorylation in differentiated SH-SY5Y cells. SH-SY5Y cells were differentiated using 10 μM retinoic acid (RA). On RA D6, cells were infected by vHCA8* with a MOI 3. At RA D9, cell cultures were treated with XE-991 or vehicle control for 20 min were collected and protein extracted for western blotting. **(A)** shows pKv7.2–7.3 phosphorylation is XE-991 dose-dependent. vHCA8*WT inhibited XE-991-induced phosphorylation at 10 μM, but vHCA8*MT did not. **(B)** shows 10 μM XE-991-induced Kv7.3 phosphorylation was inhibited by vHCA8*WT but not by vHCA8*MT treatment. *N* = 5 from 3 different cultures. Western data were normalized to β-tubulin loading standard. *** *p* < 0.001; one-way ANOVA; NS, not significant.

Treatment of SH-SY5Y cells with XE-991 (1–10 μM) produced a dose-dependent increase of pKv7.2–7.5 (Figure 8A) as detected using western blotting. SH-SY5Y cells were infected with vHCA8*WT or vHCA8*MT on RA D6. On RA D9, the cells were treated with 10 μM XE-991 for 20 min, which induced a significant increase in pKv7.2–7.5 and pKv7.3 in control and vehicle-treated cells (Figures 8A,B). XE-991 (10 μM), as compared to vHCA8*MT, PBS, or DMSO control treatments (Figures 8A,B). Phosphorylation (inactivation) of Kv7.2–7.5 induced by XE-991 treatment was inhibited by vHCA8*WT, but not vHCA8*MT pretreatment. These data are consistent with CA8*WT-mediated activation of Kv7.2–7.5 channels.

## Discussion

In this report, we show for the first time: (1) DRG transduction of primary afferent neurons with rdHSV-based vHCA8*WT gene therapy using IHC after intra-articular KJ route of vector delivery (Figures 1, 2). Double IHC staining using the V5-tag for exogenous CA8*WT showed selective vHCA8*WT uptake by DRG somatosensory neurons (advillin ~35%) and (TrkA ~46%) (Figure 2), but minimal CA8 expression after vHCA8MT (negative control) administration on the ipsilateral side (Figure 2). There was no CA8*WT expression detected in contralateral lumbar DRGs, confirming these vectors are incapable of viral replication and spread following peripheral injection. Moreover, despite the CAG promoter used, only sensory neurons (eg, overlap with advillin staining) appeared to express CA8* *ex vivo* (Figures 2A–C); and these were largely smaller TrkA expressing primary afferents (Figures 2G–I). (2) KJ injections of vHCA8*WT virus treated chronic MIA-induced OA mechanical pain with increased mechanical thresholds as compared to vHCA8*MT negative controls by D9 post-MIA (D6 after virus injection), returned to Baseline mechanical thresholds of 1.71 g by D13 post-MIA; and exceeded Baseline (analgesia) by D20 post-MIA. Mechanical thresholds achieved a maximum of 2.74 g at D56, representing significant analgesia in morphine milligram equivalents (Fu et al., 2017). (3) Motor functions were assessed by weight-bearing after MIA-induced chronic OA pain. KJ injections of vHCA8*WT virus reversed the chronic MIA-induced OA reduction in the ipsilateral lower extremity weight-bearing, in a dose- and time-dependent fashion. Weight-bearing increased in mice treated with vHCA8*WT-HD and vHCA8*WT-MD on D27 and D34, respectively, as compared to mice treated with vHCA8*MT-HD. There was a difference in weight distribution between left (OA) and right (contralateral control) hind limbs across all time points [*F*(14, 196) = 9.637, *p* = 3.898E-16] using repeated measures two-way ANOVA. Voluntary motor functions were further assessed by automated voluntary wheel running that was suppressed for 10 days (D13-post MIA), which was a significantly shorter period as compared to mechanical hypersensitivity following MIA treatment and OA chronic pain (Supplementary Figure S2). Voluntary wheel running distance was less impacted after vHCA8*WT as compared to controls (Supplementary Figure S2), and wheel running distance was restored to Baseline levels by D13, indicating no significant or lasting impact on motor function from vHCA8* treatments. (4) Parenteral administration of specific Kv7 channel inhibitor XE-991 inhibited vHCA8*WT analgesia and anti-hyperalgesia, similar to data reported by Teng et al. (2016) (Figure 5). These results suggest that vHCA8*WT mediates analgesia via decreased primary afferent excitability and reduced trafficking of nociceptive signals to the CNS associated with Kv7 channel activation that can be reversed by XE-991. (5) We also show that IA KJ injection of vHCA8*WT is associated with reduced DRG pKv7.2-5/Kv7.2 and pKv7.3/Kv7.3 as compared to vHCA8*MT treatment in this MIA-induced chronic OA pain model (Figures 6, 7). (6) Finally, we show that XE-991-induced phosphorylation (inactivation) of Kv7 channels was inhibited (channel activation) by vHCA8*WT pretreatment in differentiated SH-SY5Y cells, for Kv7.2–7.5 and less so Kv7.3 channels. Whereas, vHCA8*MT treatment produced no anti-hyperalgesia, analgesia (Figure 4), and had no effect on activation by Kv7.3 dephosphorylation at threonine-246 (Figures 6–8).

### Kv7 voltage-gated potassium channels mediate vHCA8* analgesia and anti-hyperalgesia

**Figure 9:**
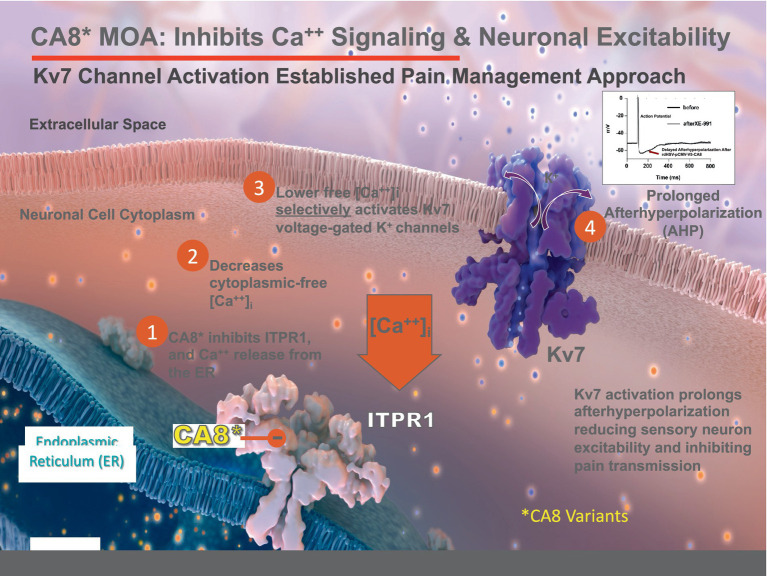
Proposed Mechanism of vHCA8* Regulation of Neuronal Excitability. CA8* is an allosteric inhibitor of ITPR1-mediated calcium release from the endoplasmic reticulum (ER) into the cytoplasm. Decreased cytoplasmic-free calcium exclusively activates Kv7 voltage-gated potassium channels to decrease neuronal excitability (see the associated manuscript, Kandel et al., in this issue). Kv7 channel activators are known in clinical practice to produce analgesia that does not depend on opioid pathways or exhibit dependence, hyperalgesia, or addiction.

Kv7 channels provide a mechanistically plausible link between reduced cytosolic free calcium, conferred by vHCA8*WT, and the resulting analgesia observed (Figure 4) (Zhuang et al., 2015; Levitt et al., 2017; Zhuang et al., 2018). KCNQ genes (KCNQ-2, −3 and –5) encode transmembrane channel proteins (Kv7.2, Kv7.3, and Kv7.5) that are known to be widely expressed as tissue-specific heterotetramers in nociceptive DRG neurons (Yu et al., 2018). Explicit evidence shows MIA-induced chronic OA pain is maintained by primary afferents in rodent models, strongly supporting our targeting primary afferent pain fibers with localized vHCA8*WT treatment which acts as a Kv7 activator (Okun et al., 2012) and suppressor of neuronal excitability (Figure 9).

All other K^+^ channels involved in attenuating neuronal excitability such as the calcium-activated K^+^ channes (Sarantopoulos et al., 2007) or the ATP-sensitive K^+^ channels (Kawano et al., 2009) are activated by elevated cytosolic Ca^2+^, this is not the case with Kv7 channels. Kv7 voltage-gated potassium channels are uniquely activated instead by lower cytosolic Ca^2+^ producing M-currents (I_M_) and reducing neuronal excitability (Marrion et al., 1991; Selyanko and Brown, 1996a,b). I_M_ exert a stabilizing effect on neuronal excitability (Brown and Passmore, 2009; Wang and Li, 2016), It is also well-established that virtually all DRG neurons express Kv7 “M”-currents and immunoreactivity (Passmore et al., 2003). Figures 6N, 7N show colocalization of Kv7.2 and Kv7.3 with neuronal marker (NeuN) confirming DRG neurons express these particular channels, Kv7 channels implicated in M-current production in sensory neurons (Cisneros et al., 2015).

These findings are highly relevant to chronic pain management because Kv7 activating therapies are highly valuable non-opioid analgesics; no available Kv7 activators are currently available for clinical use (Konishi et al., 2018; Perdigoto et al., 2018). Our proposed rdHSV-based vHCA8*WT gene therapy may provide a reasonable, more efficacious, and potentially safer localized target-specific alternative to opioids for clinical analgesia and anti-hyperalgesia.

### Great unmet need for non-opioid analgesics to treat OA

Chronic non-cancer pain is common among our population and most of these patients are inadequately treated making the development of safer analgesics is a high priority. In the absence of alternatives, long-term opioid use in treating chronic non-cancer pain has increased dramatically over the past few decades, and opioid abuse, tolerance and dependence are major public health concerns (Compton and Volkow, 2006; Paulozzi and Xi, 2008; Boudreau et al., 2009; Edlund et al., 2010a,b). In the United States, prescription opioid abuse costs alone were estimated at about $55.7 billion in 2007. Almost half this cost was attributed to lost workplace productivity, 45% to healthcare costs (e.g., abuse treatment), and 9% to criminal justice costs (Birnbaum et al., 2011). Knee OA is one of the primary causes of chronic pain, disability and increased mortality worldwide (Vitaloni et al., 2019; Cleveland et al., 2019a,b). OA pain is a major contributor to loss of quality-adjusted life-years and addressing pain and the related disability may reduce the increased mortality attributed to lower extremity OA (Cleveland et al., 2019a,b). Not surprisingly, the high disease burden of chronic lower extremity OA is accompanied by frequent opioid prescriptions (Callhoff et al., 2020).

### Monosodium iodoacetate OA preclinical model relevance

The MIA OA model produces intra-articular loss of cartilage, joint space narrowing associated with decreased weight-bearing and chronic pain (Chandran et al., 2009; Vonsy et al., 2009; Stevenson et al., 2011; Okun et al., 2012), pain-depressed wheel running and mechanical hyperalgesia in rodents, providing a useful preclinical behavioral assessment of chronic pain (Chandran et al., 2009; Vonsy et al., 2009; Stevenson et al., 2011; Okun et al., 2012). However, while weight-bearing and automated voluntary wheel running (AVWR) may only be sensitive to nonsteroidal anti-inflammatory therapeutics during the early inflammatory period which is expected through D14, we show dose-dependent improvement after vHCA8*WT treatment through D65 (Liu et al., 2011). Indeed, automated VWR was impacted primarily during the first two weeks (Supplementary Figure S2) after vHCA8*WT and not at all by vHCA8*MT exposure. This neurobehavioral response confirmed the maintenance of motor function but appeared to be less useful in monitoring analgesic responses, as compared to weight-bearing (Figure 3), and mechanically-evoked neuropathic pain in our study and studies published by others (Liu et al., 2011; Suokas et al., 2014). Systemic diclofenac treatment of MIA-induced chronic pain in animals reduces their chronic pain and improves their function short-term. Diclofenac and celecoxib can be beneficial early in the treatment of some patients with OA. These medications represent weak Kv7 channel activators (Peretz et al., 2007; Rivera-Arconada et al., 2017). The analgesia these specific medications provide could be at least partially independent of cyclooxygenase (COX) inhibition but instead may require Kv7 channel activation (Peretz et al., 2007). The critical importance of targeting Kv7 activation in primary sensory afferents in chronic OA pain is demonstrated by the dramatic short-term improvement in OA pain and function after localized intra-articular lidocaine administration (Chandran et al., 2009; Okun et al., 2012).

### Advantages of the JDNI8 rdHSV for PNS drug delivery

Latent HSV viral genomes are present as circular episomal double-stranded DNA molecules in a non-replicative state and do not express viral proteins but rather a latency-related non-coding RNA referred to as the LAT transcript (Stevens, 1987; Margolis et al., 2007). During latency, HSV-infected neurons remain unharmed by the presence of the extrachromosomal, circularized, unintegrated viral genome that is shielded from immune clearance (Wilson and Mohr, 2012). Wild-type HSV possesses the ability to reactivate from the latent state, wherein the virus begins to enter into its lytic virus replicative cycle, produces progeny virus particles that undergo anterograde transport back down the afferent fibers back to the region of the initial infection, where virus replication and the host immune response can lead to the formation of the characteristic herpes lesions.

In this study, we exploit a novel HSV-based intracellular biotherapeutics delivery system that takes advantage of the natural tropism of HSV for sensory nerves. The most advanced version of this vector system used herein is called JDNI8. JDNI8 is deleted for the host shut-off gene (UL41), which further reduces toxicity and helps provide for long-term transgene expression, as compared to prior versions. This vector is further deleted for the reiterated internal joint sequences (14 kb) flanking the unique long (U_L_) and short (U_S_) genome sequences that, together with the immediate-early (IE) gene deletions, create a vector with substantial packaging capacity (40 kb,) and the ability to accommodate large or multi-transgene payloads (Miyagawa et al., 2015). The infectivity of JDNI8 is enhanced by a double mutation in the fusion/entry envelop glycoprotein B (gB N/T). As a consequence, JDNI8 is a highly mutated replication defective (rd) form of HSV that is deleted for functional expression of all the viral IE genes (Miyagawa et al., 2015, 2017; Verlengia et al., 2017). The absence of IE gene activity prevents expression of the cascade of viral genes and thus the HSV genome is silent (Miyagawa et al., 2015). This rdHSV is unable to replicate, cause disease, harm neuronal cells, or spread to other cells. When injected into the joint, JDNI8 undergoes retrograde axonal transport to the nerve cell body in a manner like that seen with wild-type virus and persists in the cell nucleus in a latent-like state but is unable to reactivate, replicate, or induce the reactivation of potentially resident latent wild-type virus. Not surprisingly, JDNI8 requires specialized cells engineered to express the essential IE gene products in trans-on-demand for vector production consistent with a complete lack of replication (Miyagawa et al., 2015; Goins et al., 2020). In non-complementing cells, the virus genome is silent except for the engineered transgene cassette when introduced into loci (e.g., CA8* is introduced into the ICP4 locus) flanked by natural viral insulator elements to selectively maintain a localized active chromatin state within the transgene locus. This latest-generation gene therapy vector represents a major advance in the intracellular delivery of biotherapeutics like CA8*.

### Clinically relevant intra-articular route of local administration is comparable to other routes of vector administration

We show three routes of administration, including intra-dermal (RFP), intra-neural (SN), and intra-articular knee joint (KJ) injection, are comparable in transducing relevant DRG primary afferent neurons, providing evidence for the use of vHCA8* as a potential long-acting local analgesic. Localized administration of vHCA8*WT as a Kv7 activator using the intra-articular route provides several important advantages over parenteral routes. The intra-articular route is the customary, less invasive, and clinically favored route while potentially reducing the risk of inadvertent tissue exposure that will not contribute to the site of MIA-induced arthritis or may lead to off-target effects that could create safety concerns (Watson Levings et al., 2018). For example, intraneural and intra-dermal injections could potentially impact a much larger number of lower extremity sensory neurons unrelated to pain transmission, causing unwanted and unintended side effects. While DRG transduction by AAV is generally known to be inefficient when injected in a joint, we show DRG transduction for intra-articular injections of this rdHSV vector with long-lived effects.

### Past difficulties translating potential chronic pain therapeutics from animal models

A failure to translate therapeutics benefits from animal models to benefit in well-controlled human clinical trials has plagued the development of many analgesic candidates in development (Hill, 2000). A notable example is the NK1 receptor antagonist that demonstrated potent anti-hyperalgesia in animal models, but failed to show significant improvement on baseline nociception (analgesia) in human clinical studies (Hill, 2000). In our studies, we show significant dose-dependent analgesia using von Frey mechanical responses in an established OA pain model (Figure 4). These neurobehavioral responses appear to be directly dependent on vHCA8*WT treatment, as mechanical paw withdrawal thresholds were unchanged after IA KJ injections of vHCA8*MT. In addition, Kv7 channel openers have been shown to reverse mechanical allodynia and thermal hyperalgesia in rat models of inflammatory, neuropathic and bone cancer pain (McMahon et al., 1987; Mastronardi et al., 1988; Blackburn-Munro and Jensen, 2003; Erichsen et al., 2003; Passmore et al., 2003; Munro and Dalby-Brown, 2007; Wulff et al., 2009; Perdigoto et al., 2018). Further, these medications were shown in clinical practice and small-scale controlled human clinical studies to produce analgesia comparable to opioids, but without dependence, tolerance and opioid-like complications (Devulder, 2010). Establishing a likely molecular mechanism of Kv7 channel activation for CA8-induced *analgesia* and anti-hyperalgesia lends further support to the potential clinical-translational value of these findings for treating human chronic noncancer pain, given the established effectiveness of therapeutics based on this mechanism in prior human studies and clinical use. Localized administration is likely to avoid the off-target effects and other safety concerns seen previously with the systemic administration of other Kv7 activators, which greatly limited their potential clinical utility, despite potentially offering highly effective long-term analgesia.

### Summary

These data provide a strong preclinical proof-of-concept for this JDNI8-rdHSV-based CA8* gene therapy as a ‘chronic pain disease-modifying’ long-acting local analgesic. This pathway appears to provide profound, prolonged, safe analgesia and anti-hyperalgesia in this MIA-induced chronic OA pain model using the clinically relevant minimally invasive localized intra-articular route of administration.

## Materials and methods

### Animal preparations and care

All procedures related to animal use and care were preapproved by the University of Miami Institutional Animal Use and Care Committee (IACUC). All C57BL/6 J inbred male mice used in our experiments were 8–10 weeks old. Sprague Dawley (SD) rats used in experiments with neuronal DRG cultures were 2 weeks of age. All animals were housed in a facility at a controlled temperature and humidity. A 12 h light/dark cycle was provided along with water and food *ad libitum*.

### MIA-OA chronic inflammatory pain model

The osteoarthritis (OA) persistent inflammatory pain model was generated by a single intra articular (i. a.) injection of 1 mg monosodium iodoacetate (MIA, Sigma, St. Louis, MO) in 10 μL saline into the left KJ cavity using a 50 μL micro syringe with a 30-gauge needle under anesthesia by intraperitoneal (i.p.) injection of a mixture of ketamine (9 mL of 100 mg/mL), xylazine (1.8 mL of 100 mg/mL), acepromazine (3 mL of 10 mg/mL) (VEDCO, Saint Joseph, MO), and saline up to 50 mL. The mice were allowed to recover from the effect of anesthesia on the heating pad before being returned to their home cages.

### Engineering of vHCA8*WT and vHCA8*MT viruses

The WT and MT vHCA8* contructs were made by digesting the HCA8*-AAV-MCS4650 plasmids both wild-type (WT) and mutant (MT) (Goss et al., 2011; Zhuang et al., 2018), with BglII enzyme (New England Biolabs) (Upadhyay et al., 2020). Gibson Reaction (NEBuilder HiFi DNA Assembly, New England Biolabs, Ipswich, MA) was used to clone those BglII fragments upstream of a PCR fragment of T2A-GFP sequence from a glycoprotein C (gC)-T2A-eGFP fusion plasmid (Mazzacurati et al., 2015) using the following primers: T2A-GFP-F: 5-‘CTCGGTCTCGATTCTACGGAGGGCAGAGGAAGTCTGCTAACATGCGGTGACG-TCGAGGAGAATCCTGGCCCAGAGAGCGACGAGAGCGGCCT-3’, GFP-R: 5’-AGGGATGCCACCCGTAGATCT-tta-GCGAGATCCG-GTGGAGCCGG-3′. The final products from the Gibson Reaction, pAAV-CAGp-hsCA8(WT)-V5-T2A-GFP and pAAV-CAGp-hsCA8(MT)-V5-T2A-GFP were then transferred as 3473-bp NotI-digested gel isolated bands into the NotI site of ccdB^−^ pENTER 1A between the attL recombination sites to create the final products named pE-CAGp-hsCA8*(WT)-T2A-GFP and pE-CAGp-hsCA8*(MT)-T2A-GFP (Mazzacurati et al., 2015; Miyagawa et al., 2015). The LR gateway reaction using LR Clonase (ThermoFisher, Pittsburgh, PA) was used to insert the cassettes from these plasmids into JDNI8-GW41 BAC vector purified from HH8 bacteria (Miyagawa et al., 2015). Recombinants were screened by PCR across the GW cassette and confirmed by field inversion gel electrophoresis (FIGE) analysis (FIGE mapper, BioRad, Hercules, CA) of restriction enzyme digests of the recombinants (Miyagawa et al., 2015).

### Preparation, purification, and authentication of vHCA8* virus particles

The JDNI8-CAGp-V5-CA8WT-T2A-GFP (vHCA8*WT) or JDNI8-CAGp-V5-CA8MT-T2A-GFP (vHCA8*MT) vectors were produced by transfection of U2OS-4/27 complementing cells with DNA purified from BAC preps for each of the vectors. Individual isolates were purified using limiting dilution analysis and then small virus stocks were used to infect Cre-expressing ICP4/ICP27-complementing (U2OS-4/27-Cre) cells to eliminate the BAC sequences by Cre-mediated recombination (Miyagawa et al., 2015; Goins et al., 2020). Limiting dilution analyses were again performed and individual isolates lacking the BAC were identified by X-gal staining of individual plaques in 96-well plates (Thermo-Fisher, Pittsburgh, PA). Following BAC deletion, viral stocks were grown to high titer (Ozuer et al., 2002; Wechuck et al., 2002; Goins et al., 2014, 2020), and used to infect 1× 10-layer Cell Factory (Corning, Corning, NY) of U2OS-4/27 complementing cells at MOI = 0.0005 in VP-SFM MEDIA (Thermo-Fisher, Pittsburgh, PA) for 1-h at 37°C in a CO_2_ incubator. On ~D8, the CFs displayed ~90% CPE, and the next day NaCl was added to 0.45 M, and the CFs rocked for 4-h. Virus supernatant was harvested and processed by 0.8-micron CN filtration (Thermo-Fisher, Pittsburgh, PA) and subjected to centrifugation at 43,000xg for 45–90 min, followed by a dPBS wash and a second identical centrifugation step. The vector was finally resuspended in dPBS with sterile glycerol added to a final volume of 10%, and the virus was vialed in 10 μL (actual volume 12.5 μL) aliquots in cryovials and stored at -80°C. The overall titers were determined by standard plaque assay on U2OS-4/27 complementing cells (Goins et al., 2020). Aliquots of 10 μL were used for QA/QC release testing, and expression/toxicity in primary rat DRG cutulres. Toxicity was assessed by MTT assay that showed the vHCA8*WT vector was like the vHCA8*MT control vector on primary rat DRG or mock-infected DRG cultures.

### Virus transduction of DRG cell culture

DRGs were micro-dissected from 2-week-old rats, dissociated with 3 mg/mL type-I collagenase (Sigma, St. Louis, MO) in Leibovitz’s L-15 media (Thermo-Fisher, Pittsburgh, PA) for 30 min at 37°C with constant shaking, and plated on poly-D-lysine (Sigma) laminin-coated coverslips at ∼10^5^ cells per well in 24-well plates (Thermo-Fisher, Pittsburgh, PA) in 500 μL of defined Neurobasal medium with B27 supplement, Glutamax-I, Albumax-II, and P/S (Gibco/Invitrogen/Thermo-Fisher), supplemented with 100 ng/mL 7.0S NGF (Sigma). At 1–3 d post-plating, cultures were treated with 10 μM uridine and 10 μM fluorodeoxyuridine (Sigma) in the above media for 1–2 d to limit the expansion of dividing cells, such as fibroblasts and glia. Cells were then washed with PBS and incubated with NGF-supplemented Neurobasal medium as above. Virus infections were performed at D1 after plating at an apparent MOI of 5.0. Cell lysates were harvested as described elsewhere (Miyagawa et al., 2015), and western blots were probed with antibodies to CA8, V5, or β-actin (Abcam, Waltham, MA).

### HSV virus delivery

Three days later after MIA injection, male mice were anesthetized by intraperitoneal (i.p.) injection of ketamine, xylazine and acepromazine. vHCA8*WT or vHCA8*MT viral particles (10 μL of 1.4E8 PFU/mL or 1.4E6 total particles) were injected into the KJ or RFP using a 50 uL microsyringe with a 30-gauge needle. SN injections of viral particles (1.5 μL of 1.4E8 PFU/mL or 2.1E5 total particles) were made using a 35-gauge needle NanoFil syringe (World Precision Instruments, Sarasota, FL). The injection site was ~45 mm from the tip of the third toe. The needle remained at the injection site for one additional minute, before it was slowly removed to prevent leakage from the needle track.

### Immunohistochemistry

After 14 days survival times, mice were terminally anesthetized with isoflurane and perfused through the ascending aorta with saline followed by 4% paraformaldehyde (JT Baker, Fisher Scientific) with 1.5% picric acid in 0.16 M PB (pH 7.2–7.4, 4°C). After the perfusion, the L4-5 DRGs were removed and post-fixed in the same fixative for 2–4 h, then replaced with 20% sucrose overnight. Serial DRG sections (16 μm) were cut in a Leica CM1860 cryostat and processed for immunofluorescence (Jin et al., 2003). Approximately 36 sections were acquired from a single DRG. Sections from ipsilateral L4 and L5 DRG were mounted on Superfrost Plus Gold microscope slides (Thermo Scientific, #6685 L60). Eight DRG sections were read randomly per animal from four animals per treatment group (a total of 32 sections per treatment). All the sections were blocked with 2% donkey serum in 0.3% Triton X-100 for 1.5 h at RT and incubated overnight at 4°C with anti-V5 (Invitrogen) (specifically for exogenous CA8* expression, anti-chicken, 1:5000, Abcam), anti-CA8 (Santa Cruz, Santa Cruz, CA), anti-pKv7.2–7.3 (Rabbit, Biorbyt), anti-Kv7.2 (Guinea pig, Alomone), anti-pKv7.3 (Rabbit, Biorbyt), anti-Kv7.3 (Goat, MyBiosorce or Rabbit, Alomone), anti-vinculin and b-tubulin (Abcam), or anti-β-actin (Sigma). The sections were then incubated for 1 h at RT with Alexa Fluor 488-AffiniPure donkey anti-chicken secondary antibody (1:1000, Jackson ImmunoResearch lab). For double immunofluorescence, sections were incubated with a mixture of chicken V5 and Rb advillin (sensory neuronal marker, 1:500, Abcam), Rb TrkA (NGF receptor, 1:100, Abcam), overnight at 4^°^ C, followed by a mixture of Alexa Fluor 488- and 594-AffiniPure donkey secondary antibodies for 1 h at RT. The stained sections were captured with a Leica DMI 6000B fluorescence microscope. All stained DRG sections were quantified using ImageJ v1.54 for colocalization and quantification. Data were analyzed using SPSS.

### Cell culture

SH-SY5Y cells were purchased from ATCC (CRL-2266) and were cultured in a 1:1 mix of DMEM and F12 with 1% glutamax, 1% penicillin–streptomycin, 10% FBS (Invitrogen). Cells were seeded in 6-well plates with a cell density of 1 × 10^5 per well. On D1, 10 μM retinoic acid (RA, Fisher Scientific) was added to the media to differentiate SH-SY5Y cells, and the media was changed every 48 h. until D9. On D6, vHCA8 virus was loaded in cultures at a dose of MOI = 3. After the application of 10 μM XE-991 to the culture media (20 min) on D9 to cells differentiated with RA, cell cultures were collected for protein extraction with RIPA buffer plus proteinase inhibitor and phosphatase inhibitors. Western blotting was used to evaluate protein levels of exogenous CA8*, endogenous pKv7, and Kv7. DMSO was used as a vehicle control.

### Western blotting

SH-SY5Y cultures were homogenized after various treatments in RIPA buffer with a mixture of proteinase and phosphatase inhibitors. Protein samples were generally separated on 4–15% SDS polyacrylamide gels and transferred to PVDF membranes. Western blots were prepared, handled, and analyzed essentially as presented elsewhere (Zhuang et al., 2006) using anti-CA8 (Santa Cruz, Santa Cruz, CA), anti-V5 (Invitrogen), anti-pKv7.2–7.3 (Rabbit, Biorbyt), anti-Kv7.2 (Guinea pig, Alomone), anti-pKv7.3 (Rabbit, Biorbyt), anti-Kv7.3 (Goat, MyBiosorce or Rabbit, Alomone), anti-vinculin and b-tubulin (Abcam), or anti-β-actin (Sigma). Density analysis was performed using UN-SCAN-IT, standardized to β-tubulin, β-actin, or vinculin, and a one-way analysis of variance (ANOVA) was used for statistical analysis.

### Pain behavior analysis

Animals were habituated to the testing environment daily for 4 consecutive days, and Baseline collected on D5. The room temperature and humidity remained stable for all experiments. For testing mechanical sensitivity, animals were put under inverted round plastic box (Radius: 9 cm, Height: 11 cm) on an elevated metal mesh floor and allowed 60 min for habituation before the threshold test. The plantar surface of each hind paw was stimulated with a series of von Frey hairs (from 0.4 to 6 g). The threshold was taken as the lowest force that evoked a consistent brisk withdrawal response (Zhuang et al., 2006, 2015). The test was performed once a day in the first week after MIA, once every other day in the 2nd week then twice a week. The Baseline was established just prior MIA injection. The up-down method was used to calculate 50% mechanical thresholds (Dixon, 1980; Zhuang et al., 2015). All testing was performed by a trained investigator masked to treatment groups.

### Motor function was assessed using weight-bearing and automated voluntary wheel running distance

Weight-bearing distribution between the left (MIA) and right (contralateral) hind limbs were tested by the Librae Incapacitance Tester (Ugo Basile) in mice after various treatments. Data are presented as the percentage of weight distributed on the left hindlimb calculated by the formula: [weight on the left hindlimb/(weight on the left + weight on the right)] x 100. Measurements were performed before and after KJ IA MIA injection and compared with vHCA8* and with and without XE-991 administration (Helyes et al., 2010).

Automated voluntary wheel running was assessed using stainless-steel activity wheels designed for mice (diameter 23 cm; width 5 cm) with ball-bearing axles in clear polycarbonate cages (20.5 cm wide x 36.5 cm long x 14 cm high) (Bioseb) were used to measure spontaneous motor activity in mice. The wheels could be turned in either direction. Multiple activity cages were contained within a testing room. The wheels were connected to a computer that automatically recorded the distance run by each mouse in the wheel during 1-h evaluation sessions at approximately the same time of day. Mice were habituated in individual home cages for 1 session each day for 4 days. The Baseline was collected one day after the last habituation session. Mice that ran less than 275 m during Baseline measurements were removed from further evaluation. After the Baseline was obtained, the left KJ was injected with MIA (monosodium iodoacetate) to induce chronic OA pain or with saline as a control. vHCA8*WT virus was injected into the same KJ 3 days after MIA delivery. vHCA8*MT was used as a negative treatment control (Türkmen et al., 2009).

### Reagents

All chemicals or reagents were obtained from and authenticated by Sigma-Aldrich, St. Louis, MO, except as otherwise mentioned in the text.

### Quantification and statistics

To quantify immunoreactive staining of V5 for exogenous CA8, advillin, TrkA, pKv7,Kv7 and NeuN expression in the DRG, the percentages of positive neurons in the L5 and L4 DRG were determined, as described previously (Zhuang et al., 2015). The percentage of positive DRG neurons was estimated by calculating the average total number of V5-positive cells divided by the total number of NeuN-positive cells from four animals and 8 sections from each animal. Results represent 8 DRG sections read randomly from four mice in each treatment group. Quantitative evaluations were made by an investigator masked to the arrangement of DRG sections analyzed. Groups were compared using Student’s t-test, or ANOVA, followed by Fisher’s protected least significant difference test, and data presented as mean ± SEM. The criterion for statistical significance was *p* < 0.05. The sample size was *N* = 10 per group for all *in vivo* behavior experiments except as noted. IBM SPSS Statistics version 29 was used to calculate the significance between groups at each time point, incorporating a Fisher’s LSD test. The number of mice used per assay group was based on power analyses using the observed variation in prior AAV8-CA8 assays (Zhuang et al., 2015). We estimated that *N* = 8 animals per group would provide 95% power at *p* = 0.05. No animals were excluded in our analyses.

## Data availability statement

The original contributions presented in the study are included in the article/Supplementary material, further inquiries can be directed to the corresponding author.

## Ethics statement

The animal study was approved by University of Miami Institutional Animal Care and Use Committee (IACUC). The study was conducted in accordance with the local legislation and institutional requirements.

## Author contributions

GZ: Conceptualization, Data curation, Formal analysis, Investigation, Methodology, Writing – original draft, Writing – review & editing. WG: Data curation, Formal analysis, Investigation, Methodology, Writing – original draft, Writing – review & editing. MK: Data curation, Investigation, Writing – original draft, Writing – review & editing. MM: Data curation, Investigation, Methodology, Writing – original draft, Writing – review & editing. MZ: Data curation, Writing – original draft, Writing – review & editing. JG: Conceptualization, Methodology, Project administration, Writing – original draft, Writing – review & editing. YK: Data curation, Writing – original draft, Writing – review & editing. AL: Writing – original draft, Writing – review & editing. KS: Conceptualization, Investigation, Supervision, Writing – original draft, Writing – review & editing. RL: Conceptualization, Data curation, Formal analysis, Funding acquisition, Investigation, Methodology, Project administration, Resources, Supervision, Validation, Writing – original draft, Writing – review & editing.
