# Supplementary material for: Disease-modifying rdHSV-CA8* non-opioid analgesic gene therapy treats chronic osteoarthritis pain by activating Kv7 voltage-gated potassium channels
Source: Front Mol Neurosci. 2024 Jul 17;17:1416148. doi: 10.3389/fnmol.2024.1416148 (PMC11289847; doi:10.3389/fnmol.2024.1416148)
Supplement: Supplementary file 1 [file Data_Sheet_1.docx]

**Supporting Materials (Figures)**

**Supporting Figure 1.**


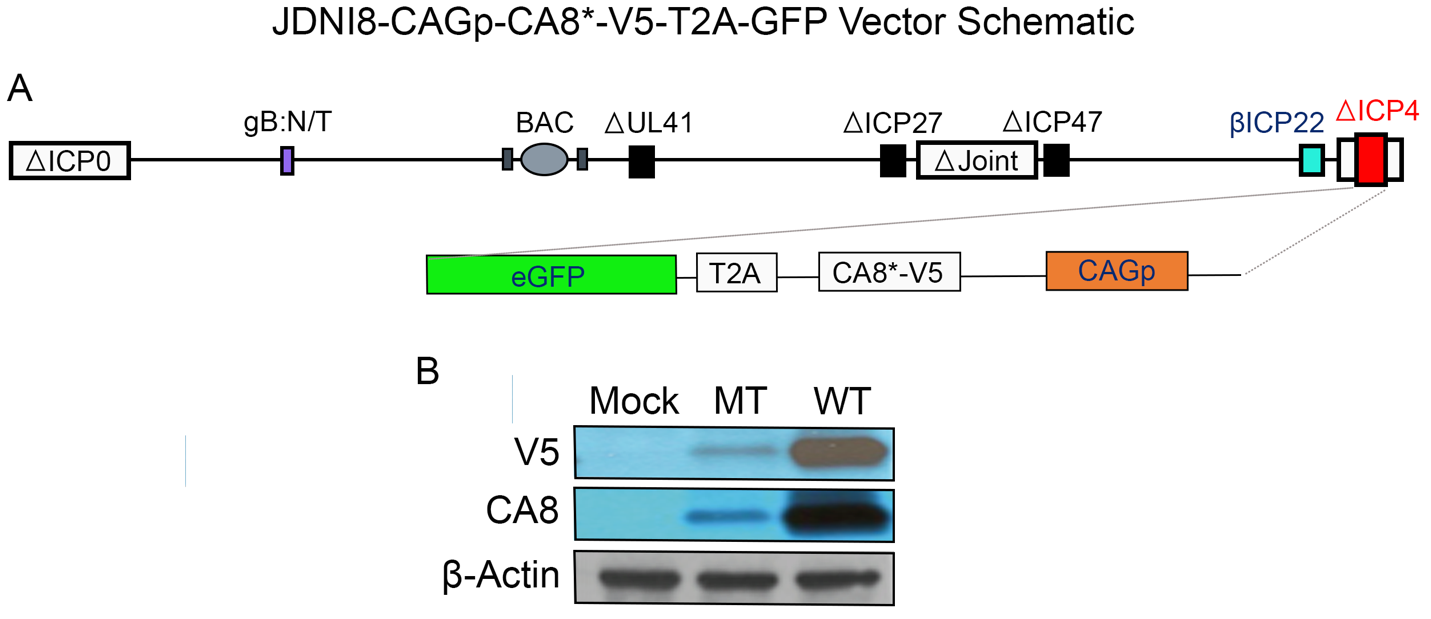


**Supporting Figure 2.**

**
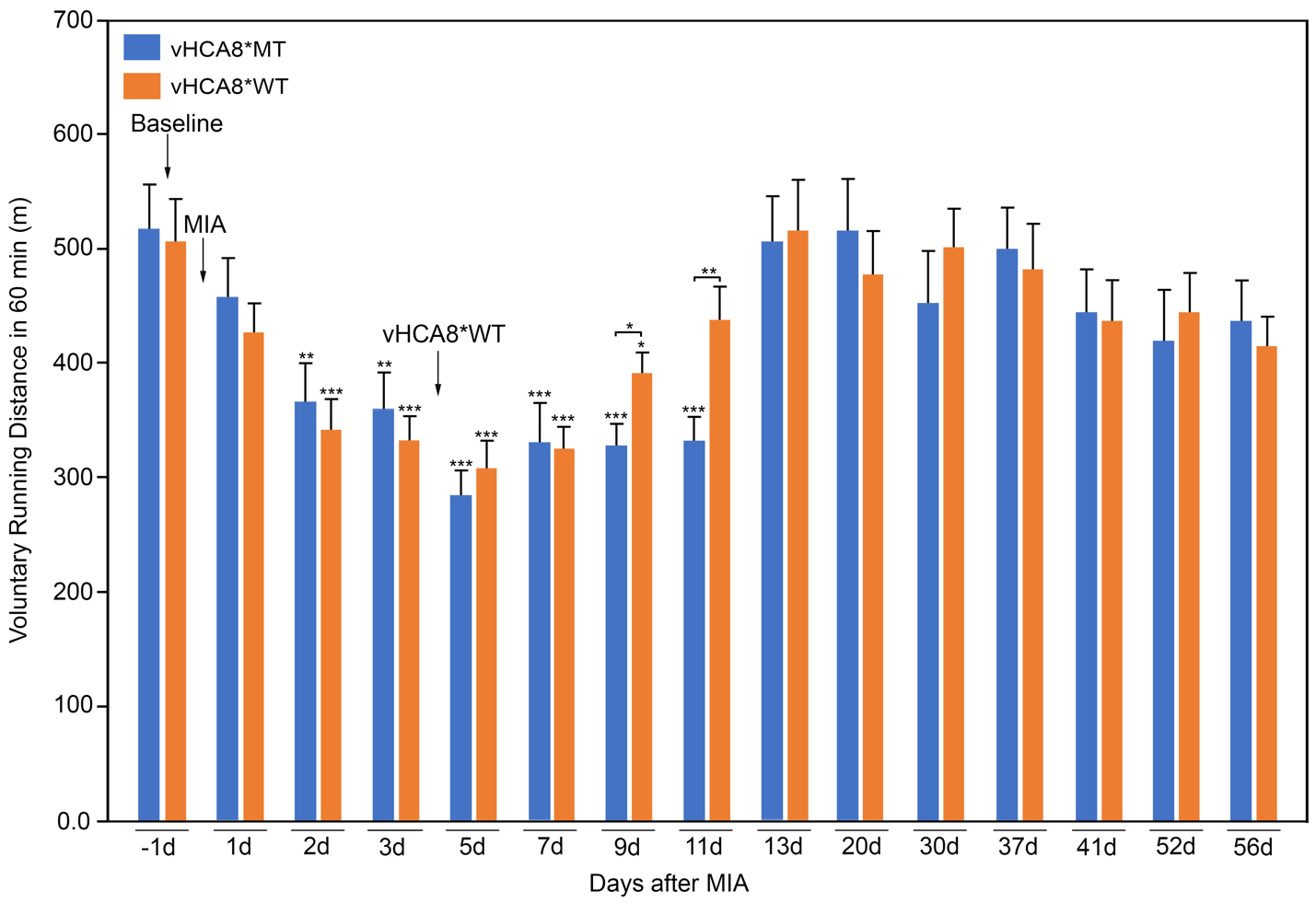
**

**Supporting Figure 3.**

**
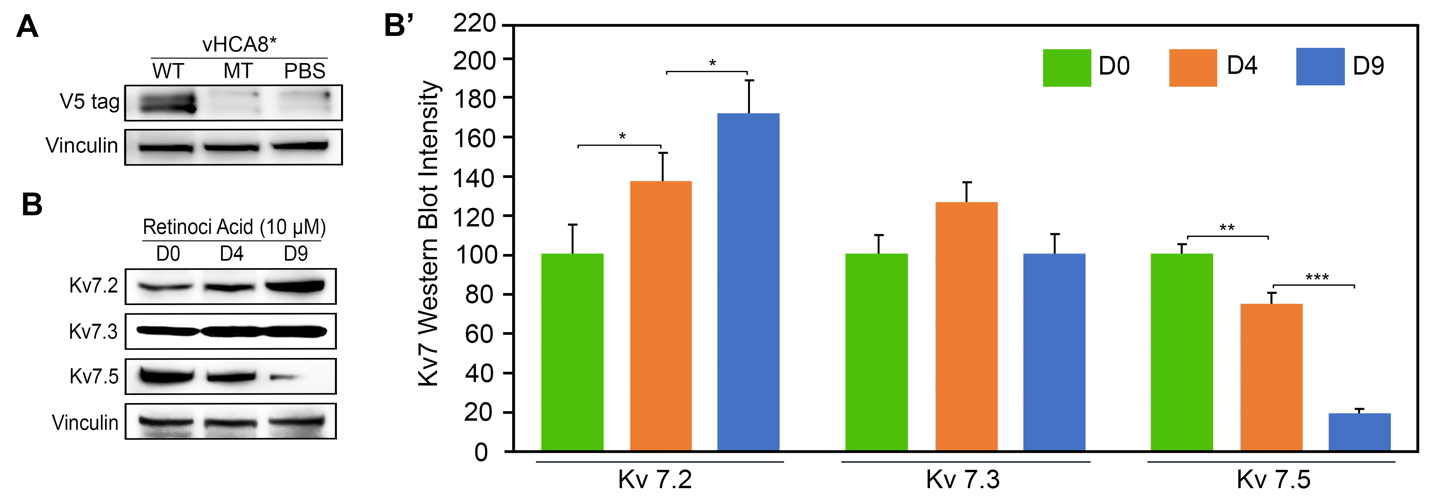
**

**Supporting Figure Legends:**

**Supporting Figure. 1. Diagram and Authentication of rdHSV Constructs in Isolated Primary Rat DRG Neuron Cultures**. Schematic of JDNI8 vector bioengineered to delete ICP0, ICP27, ICP47, ICP4, UL41, joint and incorporating into the ICP4 locus the CA8 cDNA fused to V5 (C-terminal end) and eGFP downstream of the CAG promoter. Expression in cultured dispersed primary rat DRG cells was examined at 2 days post-infection (MOI=5). Western data shows staining with anti-V5, anti-CA8 and β-Actin loading control.

**Supporting Figure. 2. Effect of vHCA8* on MIA-Induced OA Pain Repression of Voluntary Wheel Running.** MIA-treated mice exhibited a significant reduction of running distance during an hour of free access to the activity wheel from D2 - D11 in the vHCA8*MT treatment group. In the vHCA8*WT treatment group, repression in voluntary wheel running persisted for a shorter time-period (from D2 to D9) as compared to mice injected with vHCA8*MT. From D9 to D11, the vHCA8*WT injected mice showed a significant effect on anti-hyperalgesia (Fig. 4) and improvement of motor function in MIA-OA mice. These values are shown as the mean$\pm$ SEM. N=8-11. *P<0.05; **P<0.01; ***P<0.001 by Student T-test and 2-way repeated measures analysis with post hoc Fisher’s LSD test.

**Supporting Figure 3. The Relationship Between Kv7.2, Kv7.3 and Kv7.5 Expression and SH-SY5Y Differentiation by Retinoic Acid (RA).** (A) vHCA8*WT infection (D3 MOI=3) of SH-SY5Y cells shows high levels of V5 expression on western blotting as compared to vHCA8*MT on D9 of RA differentiation. (B and B’). Kv7.2 expression apparently increased with RA differentiation on D4 and D9, as compared with baseline (D0), Kv7.3 expression was unchanged with RA differentiation, and Kv7.5 expression decreased over time with RA differentiation. Vinculin was used as loading control.
